# Response of root morphology and anatomy of two alfalfa cultivars with contrasting root system architecture to phosphorus deficiency under drought stress

**DOI:** 10.3389/fpls.2026.1741660

**Published:** 2026-03-24

**Authors:** Xinya Pan, Zhi Wang, Yuzhong Fu, Delu Xu, Xianwei Wei, Pengfei Wang, Yinglong Chen, Bingcheng Xu, Gehong Wei

**Affiliations:** 1 College of Grassland Agriculture, Northwest A&F University, Yangling, China; 2 State Key Laboratory of Soil and Water Conservation and Desertification Control, Northwest A&F University, Yangling, China; 3 College of Life Sciences, Northwest A&F University, Yangling, China; 4 The UWA Institute of Agriculture, School of Agriculture and Environment, The University of Western Australia, Perth, WA, Australia

**Keywords:** alfalfa, drought, phosphorus deficiency, root anatomy, root system architecture

## Abstract

**Introduction:**

The mechanisms underlying adaptations of root system architecture (RSA) and root anatomy to phosphorus (P) deficiency and drought stress remain unclear in alfalfa (*Medicago sativa* L.). This study investigated the root response strategies of two alfalfa cultivars (WL354HQ: WL354; Longdong: LD) with contrasting RSA under P deficiency and drought, and the relationship between root traits and shoot P uptake.

**Methods:**

Root morphology and anatomy were assessed under varying P (0, 5, 20 mg P kg^-1^ soil) and water (80 ± 5% and 40 ± 5% field capacity) conditions using visual rhizoboxes, with two years field validation.

**Results:**

Under low P and drought stress within the rhizoboxes, WL354 increased fine roots in soil layers below 60 cm, maintained xylem vessel area (XVA) in lateral roots below 90 cm, enlarged cortical cell size (CCS) in taproots and 2^nd^ lateral roots across several soil layers, and reduced cortical cell files (CCF) in both taproots and lateral roots throughout all soil layers. Conversely, LD merely maintained CCS in taproots and 2^nd^ lateral roots across several soil layers, and reduced CCF in taproots across all soil layers and in 1^st^ lateral roots above 90 cm. Simple linear correlation analysis revealed that shoot P uptake was positively correlated with total root surface area in the rhizobox experiment and with xylem vessel area of the taproot in the field experiment.

**Discussion:**

Under P deficiency and drought, WL354 implemented a resource acquisition strategy by increasing resource capture and transport in deep soil, and uptake efficiency. In contrast, LD exhibited a relatively conservative strategy focused on uptake efficiency. An increase in total root surface area and xylem vessel area of the taproot may help promote shoot P uptake. These findings offer insight into root trait-based strategies for improving alfalfa productivity in low-P and drought regions.

## 1 Introduction

Alfalfa (*Medicago sativa* L.) is a perennial leguminous forage crop that provides high-quality protein and demonstrates significant ecological value through soil conservation and water retention (Li et al., 2020). It is a cornerstone species in the establishment of artificial grasslands in the semiarid and arid agroecosystems of China (Gu et al., 2018). However, its productivity is increasingly threatened by climate change-induced drought intensification (Cook et al., 2014) and reduced phosphorus (P) bioavailability (Goll et al., 2018), both of which critically limit alfalfa productivity and forage quality (Zhang et al., 2020).

Root functional traits are key determinants of plant resource acquisition efficiency and ecosystem services (Freschet et al., 2021). Root system architecture (RSA)—the three-dimensional spatial configuration and topological organization of roots—plays a critical role in plant resource acquisition efficiency and stress resilience (Liu et al., 2021; Shao et al., 2021). In alfalfa, RSA modulates symbiotic nitrogen fixation capacity, water and nutrient uptake, and drought adaptation, thereby governing yield stability (Bucciarelli et al., 2021). Under drought stress, plants exhibit depth-foraging plasticity by developing vertically oriented roots to exploit deep soil moisture (Lozano et al., 2020; Zhan et al., 2015). Conversely, under low-P conditions, shallow, highly branched root systems enhance P capture by increasing surface soil exploration (Sun et al., 2018). Strategic P fertilization induces adaptive RSA remodeling, promoting deeper root proliferation and improving hydraulic redistribution capacity, which enhances both water-use efficiency and drought tolerance (Fan et al., 2015; Zhang et al., 2020). These RSA-mediated trade-offs between hydrological and nutritional foraging highlight the necessity for genotype-specific management in water-limited, P-deficient environments.

Root anatomical traits are crucial for understanding root functions and the linkages between architecture and function (Zhou et al., 2022). Root anatomy influences the lateral and vertical transport of water and nutrients within roots and is a major determinant of the metabolic cost associated with root construction and maintenance (De Bauw et al., 2019; Gambetta et al., 2013; Lynch et al., 2021). Under various edaphic stresses, anatomical adjustments reflect root system adaptation strategies (Lynch, 2019). Larger and more numerous xylem vessels may enhance root water absorption when soil moisture is sufficient. However, reducing root xylem vessel diameters can be an advantageous water-saving strategy under drought stress, despite reducing axial hydraulic conductivity (Prince et al., 2018; Richards and Passioura, 1989). Reduced cortical area in maize roots lowers the metabolic burden and increases yield in low-P environments (Galindo‐Castañeda et al., 2018). Similarly, under low-P and drought stress, the number of cortical cell files decreases in two rice cultivars (NERICA4 and NERICA-L-19), reducing root metabolic costs (De Bauw et al., 2019).

Phenotypic analysis of RSA in soil is challenging due to soil heterogeneity and opacity (Burridgea et al., 2016). Traditional static and destructive root sampling methods limit dynamic observations of root growth (Fan et al., 2015). Additionally, most studies have mainly concentrated on the effects of drought or low P on alfalfa root morphology, with limited research exploring the link between root morphology and anatomy under different water and P supply conditions (He et al., 2017). Rhizoboxes, which enable non-destructive and in situ observation of root growth through transparent panels, simulate the soil growth environment and overcome the limitations of destructive sampling and the inability to dynamically observe roots (Bucciarelli et al., 2021). This study integrated rhizoboxes with field experiments under varying soil moisture and P supply conditions. The main objectives of this study were: (1) to investigate the root morphological and anatomical adaptive strategies of two alfalfa cultivars with contrasting RSA in response to P deficiency under drought stress; (2) to evaluate the impacts of changes in root traits on shoot P uptake and growth. This research enhances the understanding of key root traits involved in water and P absorption in alfalfa, potentially identifying candidate traits for breeding alfalfa with improved drought and low P tolerance.

## 2 Materials and methods

### 2.1 Plant materials

Based on our previous research results, WL354HQ (WL354) originated from the United States, and Longdong (LD) alfalfa originated from China with contrasting RSA were selected (Pan et al., 2023). Under normal growing conditions at the seedling stage, WL354 had a deeper root system and a greater number of lateral roots. In contrast, LD had a shallower root system and fewer lateral roots (**Supplementary Figure 1**). The seeds of WL354 and LD were obtained from Barenbrug International Grass Industry Co., Ltd (Tianjin, China) and the Gansu Institute of Grassland Ecology, respectively.

### 2.2 Rhizobox experiment

#### 2.2.1 Experimental design and growth conditions

The experiment was carried out from May to September 2021 in an outdoor rain shelter at the State Key Laboratory of Soil and Water Conservation and Desertification Control in Yangling (34°16’N, 108°4’E), Shaanxi Province, China. A visualization rhizobox cultivation system with dimensions of 30 × 5 × 150 cm (length × internal width × depth) was employed (**Supplementary Figure 2**). This rhizobox consisted of a white polyvinyl chloride foam board on one side and a transparent polycarbonate board on the other side. The transparent polycarbonate board had fixed grooves on both sides, through which black light-blocking panels could be inserted to prevent root exposure to light. The rhizoboxes were placed at a 45° angle to the ground, with the transparent board facing downward to maximize the visibility of the root system on the imaged transparent side (Bontpart et al., 2020).

An aeolian sandy soil with the following properties: total N of 8 mg kg^-1^; available P of 2 mg kg^-1^; field capacity of 16.6%; and pH of 8.49 was used for the experiment. The soil was air-dried and passed through a 2-mm sieve. Two water treatments were implemented: well-watered (80 ± 5% field capacity, HW) and drought stress (40 ± 5% field capacity, LW) (Fan et al., 2015). Three P supply levels of 0, 5, and 20 mg P kg^-1^ dry soil (hereafter referred to as P0, P5, and P20, respectively) were established using NH_4_H_2_PO_4_ (He et al., 2017). N in the low-P treatment was supplemented with CH_4_N_2_O to match the N input of the sufficient P treatment. Each rhizobox was filled with 25.5 kg of soil, with approximately 5 cm remaining from the soil surface to the top of the box. For every 1 kg of dry soil, 2.2 mg of N, 2.3 mg of K, 0.48 mg of Mg, 1.6 mg of Ca, and 0.64 mg of S were mixed into the soil as a basal fertilizer. The fertilizer was uniformly mixed with the soil and packed into the rhizoboxes. Seven days after the rhizoboxes were filled with soil, the soil was irrigated to 80% of the field capacity, and seeds were sown after the water had penetrated the soil. Healthy alfalfa seeds with uniform size were disinfected and rinsed (Wang et al., 2015), and then sown near the transparent polycarbonate board side about 2 cm below the soil surface in the rhizoboxes. Seedlings were thinned to leave one plant per rhizobox when they reached approximately 5 cm in height, and plants of similar size were retained in each replication. There were three biological replicates in each treatment for a total of 36 rhizoboxes. During the seedling stage, the plants were adequately watered. Drought stress was initiated at the branching stage (70 days after sowing). The soil water content was measured and controlled using the weighing method. The rhizoboxes were weighed and watered every two days at 18:00. The positions of the rhizoboxes were exchanged weekly. After 50 days of drought treatment (120 days after sowing), when the first plant reached the bottom of the rhizobox, the plants were harvested. Simultaneously, approximately one-fifth of the plants had their root systems reaching the bottom of the rhizoboxes.

#### 2.2.2 Sampling and measurements

On days 0, 9, 15, 30, and 50 following the application of drought, roots were traced on the transparent polycarbonate board using different colored markers to monitor the dynamic growth of the roots (**Supplementary Figure 2**). The newly emerged roots were traced on transparent films. Subsequently, these films were scanned in greyscale at a resolution of 300 dpi using an Epson desktop scanner (V800, Long Beach, USA). The obtained images were analyzed using WinRHIZO Pro (v2009, Regent Instruments, Montreal, Canada) to acquire the total root length. At the same time, the maximum root depth was measured using a ruler, and the number of root tips was counted manually.

At harvest, the shoots were severed from the roots. Beginning with the upper soil layer of the rhizoboxes, the root system was divided into four sections: the 0–30 cm layer, the 30–60 cm layer, the 60–90 cm layer, and the layer below 90 cm. For each section, a 1 cm long segment of the root was cut and preserved in a formaldehyde-acetic acid-ethanol fixative (FAA, comprising 75% ethanol, glacial acetic acid, and 40% formaldehyde), and then stored at 4 °C for subsequent paraffin sectioning (Freschet et al., 2021). Taproots from the middle section were selected, and the 1^st^ order lateral roots were taken at a distance of 5 cm from the root base, and the 2^nd^ order lateral roots were taken at a distance of 2 cm from the root tip. The root classification was shown in **Supplementary Figure 1** (Clémen et al., 2022). After dehydration using a series of alcohol solutions, these root segments were embedded in paraffin individually (Freschet et al., 2021). Subsequently, the roots were sliced into 5 μm thick sections using a LEICA automatic microtome. The slices were fully dried and stained with toluidine blue. Images of the slices were captured using a MoticBA410 optical microscope at a 4× magnification with an additional 0.65× adapter, and were saved using Motic Images Advanced 3.2 software. ROOTSCAN 2.4 software was used to analyze the images to obtain data on root anatomical traits, including total stele area, xylem vessel area, total cortical area, cortical cell files, and cortical cell size (Burton et al., 2012). A few taproots that could not be completely captured under the optical microscope and could not be analyzed by ROOTSCAN 2.4. For these cases, we used a fluorescence microscope to capture images and analyzed them using ImageJ software, and the cortical cell files were counted manually (Zhou et al., 2020). Total stele area to total cortical area ratio = total stele area/total cortical area.

Root segments from different soil layers were collected, and washed with deionized water and carefully placed on transparencies without overlapping. Subsequently, the transparencies were scanned in greyscale at a resolution of 300 dpi using the desktop scanner. The acquired images were analyzed with WinRHIZO Pro to obtain diverse root morphological characteristics, including average root diameter, root length, and root length in two diameter classes (Qiao et al., 2019). It should be noted that root diameters less than 0.2 mm are classified as fine roots in herbaceous plants (Bergmann et al., 2017). In this study, roots with a diameter < 0.25 mm were classified as fine roots, while roots with a diameter ≥ 0.25 mm were classified as coarse roots (Pan et al., 2023). The number of root tips in different soil layers was counted manually. The overall root diameter was determined as the average of the root diameters in each soil layer. The total root length, total surface area, fine root length, and coarse root length were calculated as the sum of the corresponding traits in each soil layer. Fine root length to coarse root length ratio = fine root length/coarse root length.

Root subsamples from the same plant were combined into one root sample. The root and shoot samples were dried in an air-forced oven at 75 °C for 72 h to determine root and shoot dry mass. The dried root and shoot samples were ground using a high-speed grinder (MM400, Retsch, Germany) and then digested with concentrated H_2_SO_4_-H_2_O_2_. The total P concentration was measured by a molybdenum-antimony colorimetric method (Grimshaw et al., 1989). Shoot/root P uptake = shoot/root dry mass × P concentration in shoot/root (Wu et al., 2021).

### 2.3 Field experiment

#### 2.3.1 Experimental design and growth conditions

The field experiment was conducted at the experimental site of the Sand Control Institute in Yulin (38°19’N, 109°42’E), Shaanxi Province, China. This region is characterized by a temperate, arid, and semi-arid continental monsoon climate. The monthly mean temperature and precipitation data for 2019, 2020, and 2021 are shown in **Supplementary Figure 3**. Rainfall is mainly concentrated from July to September. The elevation of the site is 1080 m, and the average annual total radiation is 139.23 kJ cm^-2^, with an average of 2815 h of sunshine annually. The area features abundant solar radiation with few cloudy days. The soil properties at the experimental site were identical to those used in the rhizoboxes.

The experiment was designed using a two-factor randomized block design. Water treatments were set as well-watered (600 mm yr^-1^, HW) and drought stress (300 mm yr^-1^, LW). These treatments were applied from May 2020 to August 2021 and delivered through drip irrigation. There were two P supply levels, high P (13 g m^-2^ yr^-1^, HP) and low P (1.5 g m^-2^ yr^-1^, LP), with P fertilizer applied in the form of superphosphate to the soil (Gu et al., 2018). Each treatment was replicated three times for each of the two cultivars, resulting in a total of 24 plots. The size of each plot was 2.5 m × 2.5 m. Sowing was carried out in June 2019 using manual row seeding, with 7 rows in each plot and a row spacing of 30 cm. The sowing depth was 1–2 cm, and the seeding rate was 6.4 g m^-2^. Watering and weeding were conducted periodically. Mowing was carried out in July and August of 2020 and 2021. The annual forage yield was calculated as the sum of the biomass in July and August of 2021. At the mowing in August 2021, root traits and P uptake were measured.

#### 2.3.2 Sampling and measurements

A healthy plant with uniform growth was excavated from each plot using a shovel. The excavated soil block was a 30 cm × 30 cm square with a depth of 30 cm. The soil block containing the roots was separated from the surrounding soil and washed gently with deionized water. A 1 cm long taproot from the middle section and a 1 cm long lateral root located 5 cm from the root base were cut. These roots were preserved in a FAA fixative for the preparation of paraffin sectioning. The roots were stored in an icebox during transportation to the laboratory and maintained at 4 °C until paraffin sections were made. The methods for preparing and analyzing root morphological and anatomical traits were the same as those described in section 2.2.2.

For each plot, two rows with even growth were chosen. In each row, 30 cm long strips of alfalfa were cut, with a 5 cm stubble remaining. After drying, the shoots were weighed. The roots were scanned first and then dried and weighed. The P concentration of the shoot was measured. The methods for determining and calculating shoot P uptake were the same as those described in section 2.2.2.

### 2.4 Statistical analysis

Statistical analysis was conducted using SPSS 21.0 (IBM Corp, Armonk, USA). Three-way ANOVAs were performed for the rhizobox and field experiments to examine the influences of genotype, water, P, and their interactions on all traits. When the three-way ANOVA indicated significant main effects of genotype, water, or P, differences between the two cultivars and two water treatments were analyzed by independent samples t-tests, and differences among the three P levels were determined by Tukey’s HSD *post hoc* test within their respective treatment groups. Specific pairwise t-tests conducted to interpret significant differences were treated as exploratory results. To avoid the impact of multicollinearity among root traits, principal component (PC) analysis was performed separately on root morphological and anatomical traits. The root traits with the largest absolute loading in each PC were selected for Pearson correlation analysis (Wang et al., 2026). If the selected root morphological or anatomical traits were significantly positively correlated, the trait with the product of its largest absolute loading and the corresponding PC’s contribution ratio was retained for simple linear regression analysis to examine its relationship with shoot phosphorus uptake. All the root traits in the rhizoboxes were scale-standardized when conducting an overall assessment of root traits adaptive strategies. All data from the rhizobox and field experiments were analyzed separately. Origin 2018 (OriginLab, Northampton, Massachusetts, USA) software was used for graphing. The value for each trait was the average of three biological replicates and was presented as the mean ± standard error in the figures. The abbreviations of the traits were listed in **Supplementary Table 1**.

## 3 Results

### 3.1 Response of root morphological traits to water and P treatments in the rhizoboxes

#### 3.1.1 Whole root morphology

**Figure 1:**
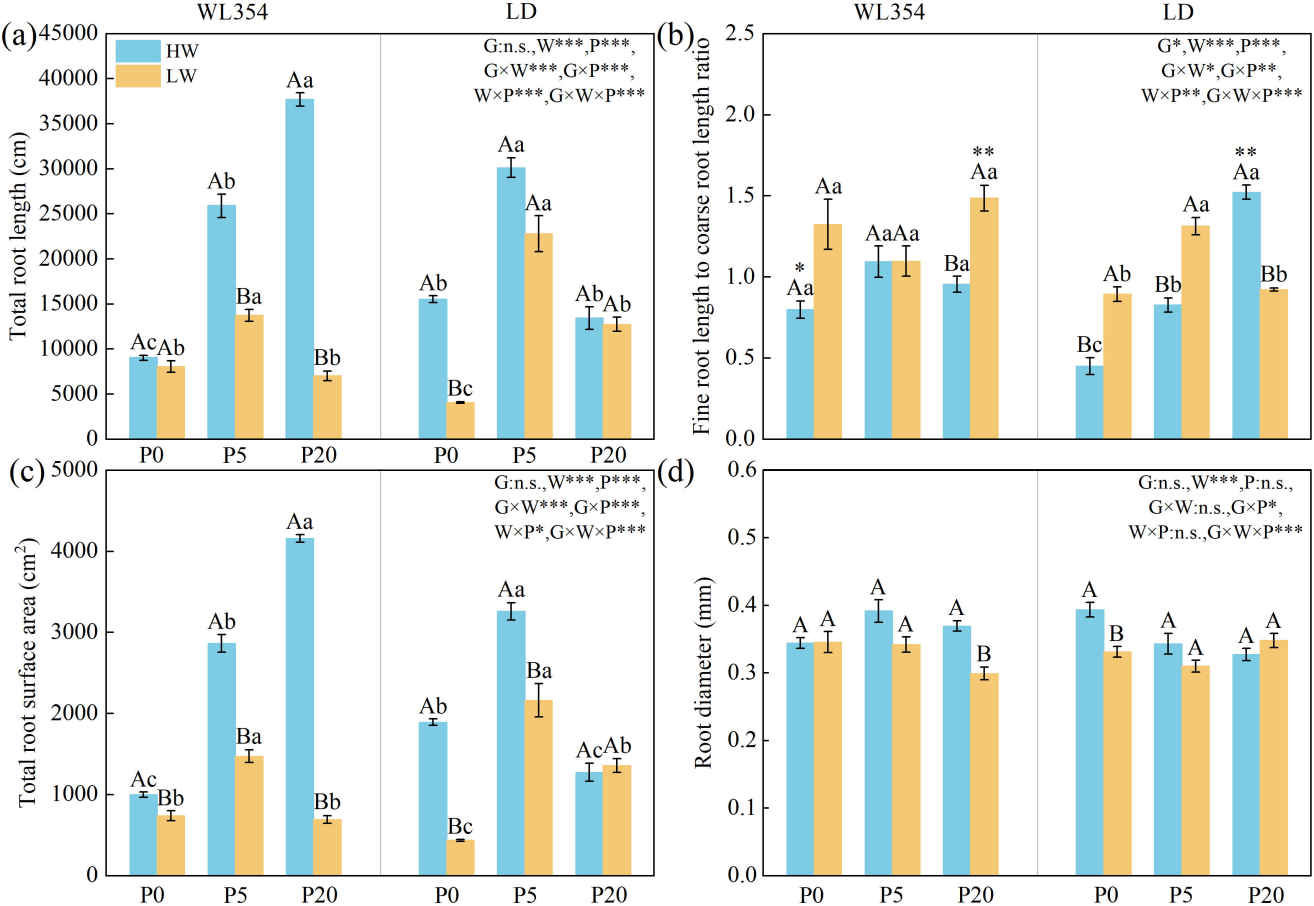
Total root length **(a)**, fine root length to coarse root length ratio **(b)**, total root surface area **(c)**, and root diameter **(d)** of WL354 and LD alfalfa under different water and phosphorus treatments in the rhizoboxes. Data represented as mean ± SE (n=3). W, water; P, phosphorus; G, genotypes; *P < 0.05; **P < 0.01; ***P < 0.001; n.s, no significant in the three-way ANOVA. When the three-way ANOVA indicated significant main effects of genotype, water, or P, differences within their respective treatment groups were analyzed. Different uppercases indicate significant differences between HW and LW treatments under the same genotypes and phosphorus treatments determined by independent samples t-test (*P* < 0.05); different lowercases indicate significant differences among P0, P5, and P20 treatments under the same genotypes and water treatments according to Tukey’s HSD *post hoc* test (*P* < 0.05); *, ** and *** above the columns indicate significant difference between WL354 and LD under the same water and phosphorus treatments determined by independent samples t-test (*P < 0.05, **P < 0.01, ***P < 0.001). HW, well-watered; LW, drought stress; P0, P5, and P20 refer 0, 5, and 20 mg P kg^-1^ dry soil.

Except for genotype, other factors significantly impacted total root length and total root surface area, and all factors significantly affected the fine root length to coarse root length ratio (**Figures 1a–c**). In the two alfalfa cultivars, water, genotype × P interaction, and genotype × water × P interaction significantly influenced root diameter (**Figure 1d**). Compared to HW conditions, the LW treatment significantly reduced total root length, total root surface area, and root diameter in both cultivars across most P levels. As P levels increased, the reductions in these traits were more pronounced in WL354, but less so in LD. Under LW conditions, total root length and total root surface area showed consistent responses to increasing P levels, peaking at P5 for both cultivars. Notably, the fine root length to coarse root length ratio in WL354 remained relatively high under LW and P0 conditions, nearly matching the values under HW and P20 conditions.

#### 3.1.2 Root morphology across various soil layers

**Figure 2:**
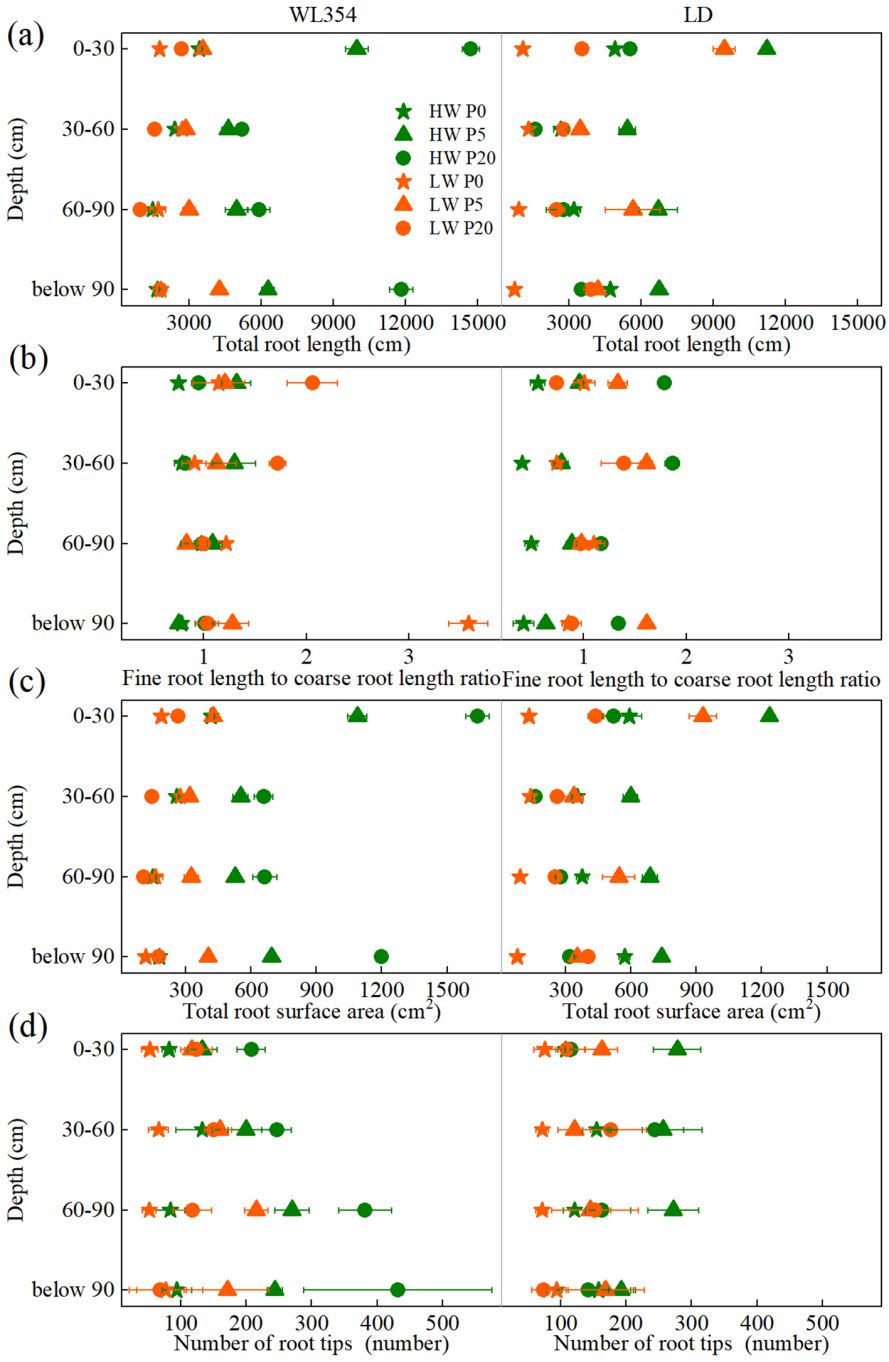
Total root length **(a)**, fine root length to coarse root length ratio **(b)**, total root surface area **(c)**, and number of root tips **(d)** of WL354 and LD alfalfa in each soil layer under different water and phosphorus treatments in the rhizoboxes. Data represented as mean ± SE (n=3). HW, well-watered; LW, drought stress; P0, P5, and P20 refer 0, 5, and 20 mg P kg^-1^ dry soil.

According to three-way ANOVA, water and P treatments significantly influenced total root length, fine root length to coarse root length ratio, total root surface area, and number of root tips in each soil layer, except for the fine root length to coarse root length ratio in the 60–90 cm soil layer (**Supplementary Table 2**). Compared to HW conditions, the LW treatment significantly reduced total root length, total root surface area, and number of root tips in each soil layer for both cultivars across most P levels (**Figures 2a, c, d**). As P levels increased, these reductions became more pronounced in WL354 but less so in LD. An exception was observed in the number of root tips in LD, where the reduction initially decreased and then increased across soil layers. Regarding the fine to coarse root length ratio, the LW treatment elicited specific responses (**Figure 2b**). In WL354, the LW treatment significantly increased it in the soil layers above 60 cm at P20 and in the soil layers below 60 cm at P0. In LD, the LW treatment significantly increased it at P0 and P5 but decreased it at P20 across all soil layers. Under LW conditions, the total root length, total root surface area, and number of root tips in each soil layer of both WL354 and LD reached the maximum values at P5. However, the variation trend for the fine to coarse root length ratio diverged, with WL354 peaking at P20 in the soil layers above 60 cm and peaked at P0 in the soil layers below 60 cm, whereas LD peaked at P5 across all layers.

#### 3.1.3 Dynamic changes in root morphology

**Figure 3:**
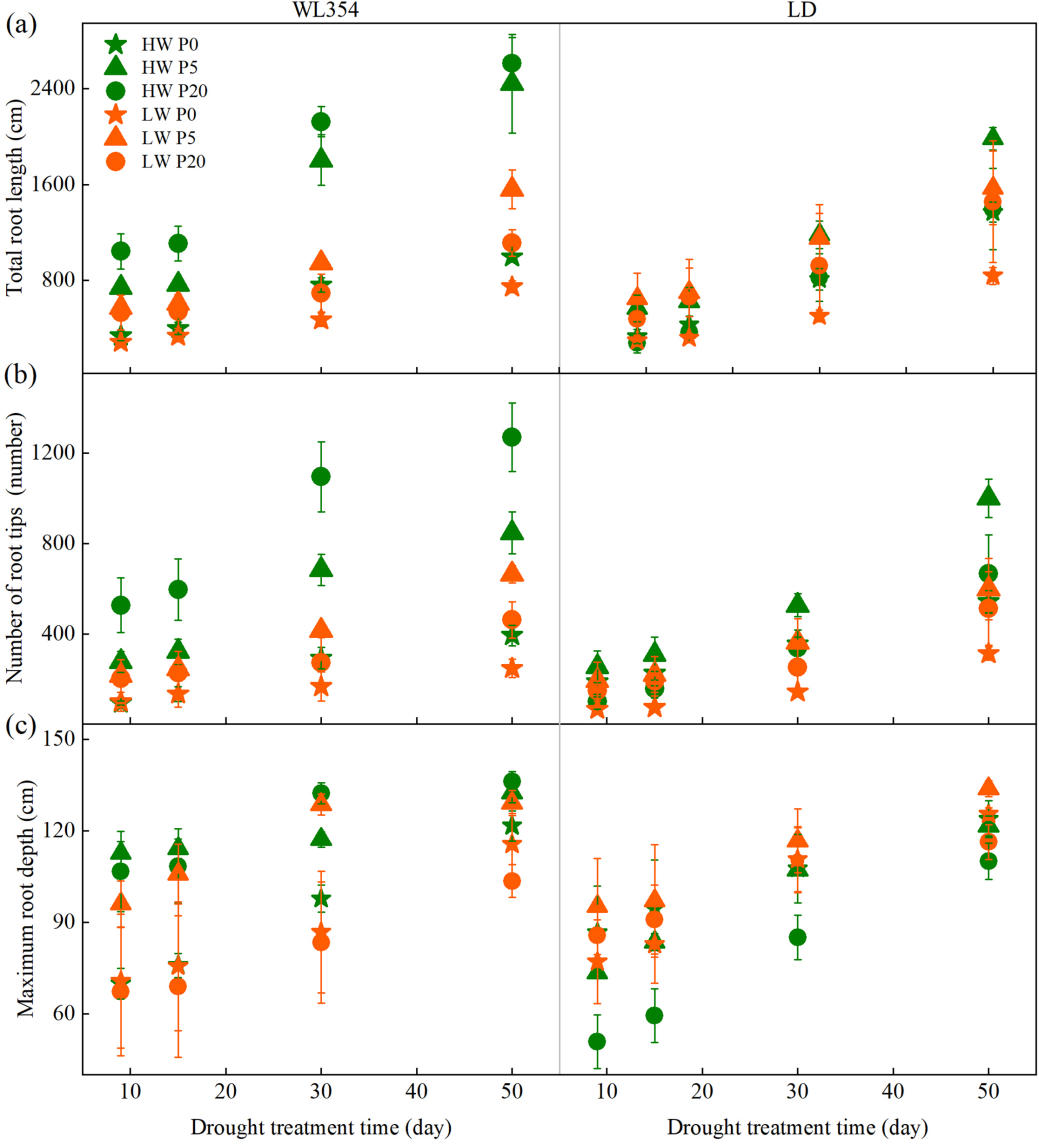
Dynamic changes in total root length **(a)**, number of root tips **(b)**, and maximum root depth **(c)** of WL354 and LD alfalfa under different water and phosphorus treatments in the rhizoboxes. Data represented as mean ± SE (n=3). HW, well-watered; LW, drought stress; P0, P5, and P20 refer 0, 5, and 20 mg P kg^-1^ dry soil.

Water treatments significantly affected total root length and the number of root tips on the 30^th^ and 50^th^ day of drought stress. P treatments significantly affected total root length and number of root tips throughout the entire drought period, and maximum root depth on the 50^th^ day (**Supplementary Table 3**). The LW treatment reduced total root length, number of root tips, and maximum root depth in WL354 across most P levels (**Figure 3**). These reductions in total root length and number of root tips intensified, whereas the reduction in maximum root depth diminished with drought duration. The LW treatment mainly reduced total root length and number of root tips in LD at P0 and P5, with the reduction intensified with drought duration. In contrast, the LW treatment increased maximum root depth in LD at P20, with the increment diminished with drought duration. Under LW conditions, total root length, number of root tips, and maximum root depth were highest at P5, and total root length and number of root tips were lowest at P0 for both WL354 and LD. Although the differences in these three traits of the two cultivars caused by P intensified with drought duration, the magnitude of these differences was lower than that caused by the LW treatment. This indicated that as drought duration extended, the impact of drought on root morphology became the dominant factor, outweighing the influence of P supply.

### 3.2 Response of root anatomical traits to water and P treatments in the rhizoboxes

#### 3.2.1 Total stele area to total cortical area ratio and xylem vessel area

**Figure 4:**
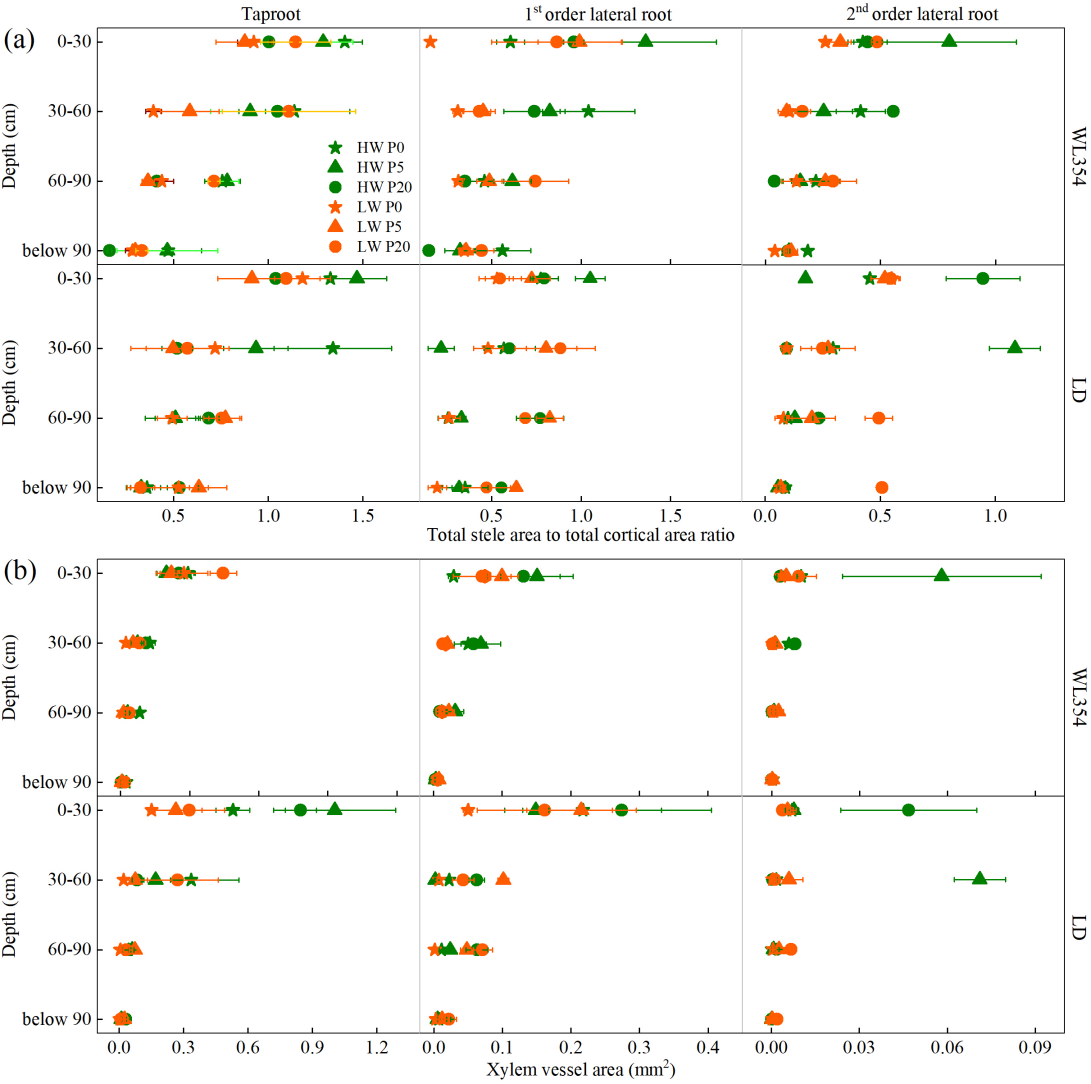
Total stele area to total cortical area ratio **(a)**, and xylem vessel area **(b)** of WL354 and LD alfalfa in each soil layer under different water and phosphorus treatments in the rhizoboxes. Data represented as mean ± SE (n=3). HW, well-watered; LW, drought stress; P0, P5, and P20 refer 0, 5, and 20 mg P kg^-1^ dry soil.

The results of the three-way ANOVA of SCR and XVA in each soil layer under different water, P and genotype treatments in the rhizoboxes were presented in **Supplementary Table 4**. Under the combined LW and P0 treatment, WL354 exhibited a generalized reduction in SCR across almost all taproots and lateral roots compared to other treatments (**Figure 4a**). For LD, the SCR of lateral roots remained consistently low across soil layers under the combined LW and P0 treatment compared to other treatments, while the SCR of taproots in the 0–60 cm layers was highest at P0 compared to the other P levels under LW conditions.

The influence of water and P treatments on XVA was depth-dependent and cultivar-specific (**Figure 4b**). In WL354, significant treatment effects were confined to the 0–30 cm soil layer, whereas in LD, the effects extended to the 0–60 cm soil layers. Notably, in soil layers below 60 cm, XVA for both cultivars under the combined LW and P0 treatment showed no significant variation compared to other treatments.

#### 3.2.2 Cortical cell size and cortical cell files

**Figure 5:**
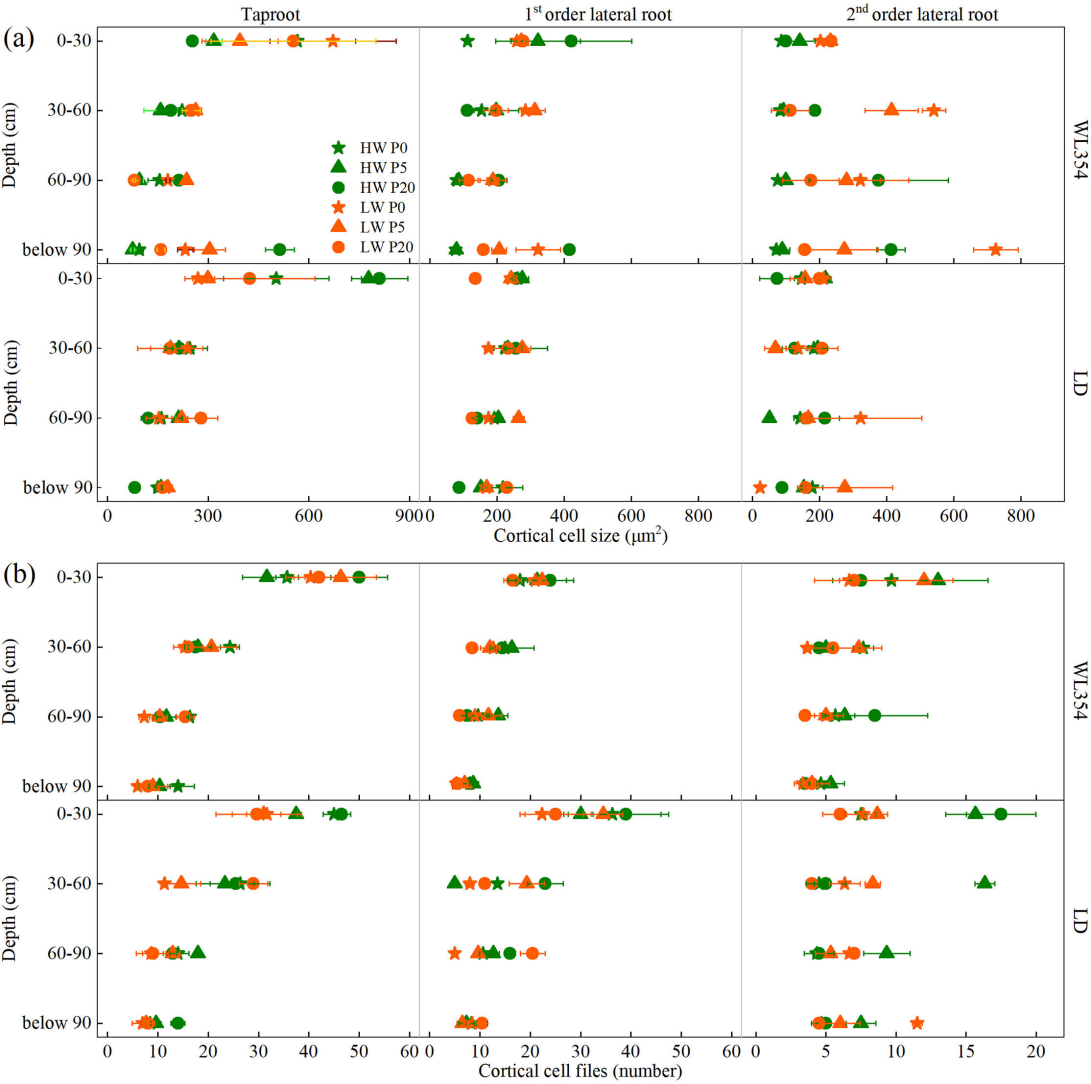
Cortical cell size **(a)**, and cortical cell files **(b)** of WL354 and LD alfalfa in each soil layer under different water and phosphorus treatments in the rhizoboxes. Data represented as mean ± SE (n=3). HW, well-watered; LW, drought stress; P0, P5, and P20 refer 0, 5, and 20 mg P kg^-1^ dry soil.

The results of the three-way ANOVA of CCS and CCF in each soil layer under different water, P and genotype treatments in the rhizoboxes were presented in **Supplementary Table 4**. Under the combined LW and P0 treatment, CCS of the taproots and 2^nd^ lateral roots of both cultivars remained relatively high across different soil layers (**Figure 5a**). For WL354, taproots in the 0–30 cm soil layer, and 2^nd^ lateral roots in the 30–60 cm and below 90 cm soil layers showed markedly higher CCS than those in other treatments. And taproots in the 30–60 cm soil layer showed no marked difference from other treatments. For LD, the CCS of taproots in 30–60 cm and below 90 cm layers, as well as 2^nd^ lateral roots in the 0–30 cm soil layer, showed no marked difference compared to other treatments. However, the CCS of its 2^nd^ lateral roots in the 60–90 cm soil layer was markedly higher than in other treatments.

Regarding CCF, WL354 showed a generalized reduction under the combined LW and P0 treatment, with values across almost all root orders and soil layers being lower than those in other treatments (**Figure 5b**). LD exhibited a root-order-dependent response. While CCF for its taproots across all layers and 1^st^ lateral roots in the soil layers above 90 cm were generally lower than those in other treatments, the CCF of its 2^nd^ lateral roots remained higher.

### 3.3 Overall assessment of root traits response characteristics in WL354 and LD alfalfa to P deficiency under drought stress in the rhizoboxes

**Figure 6:**
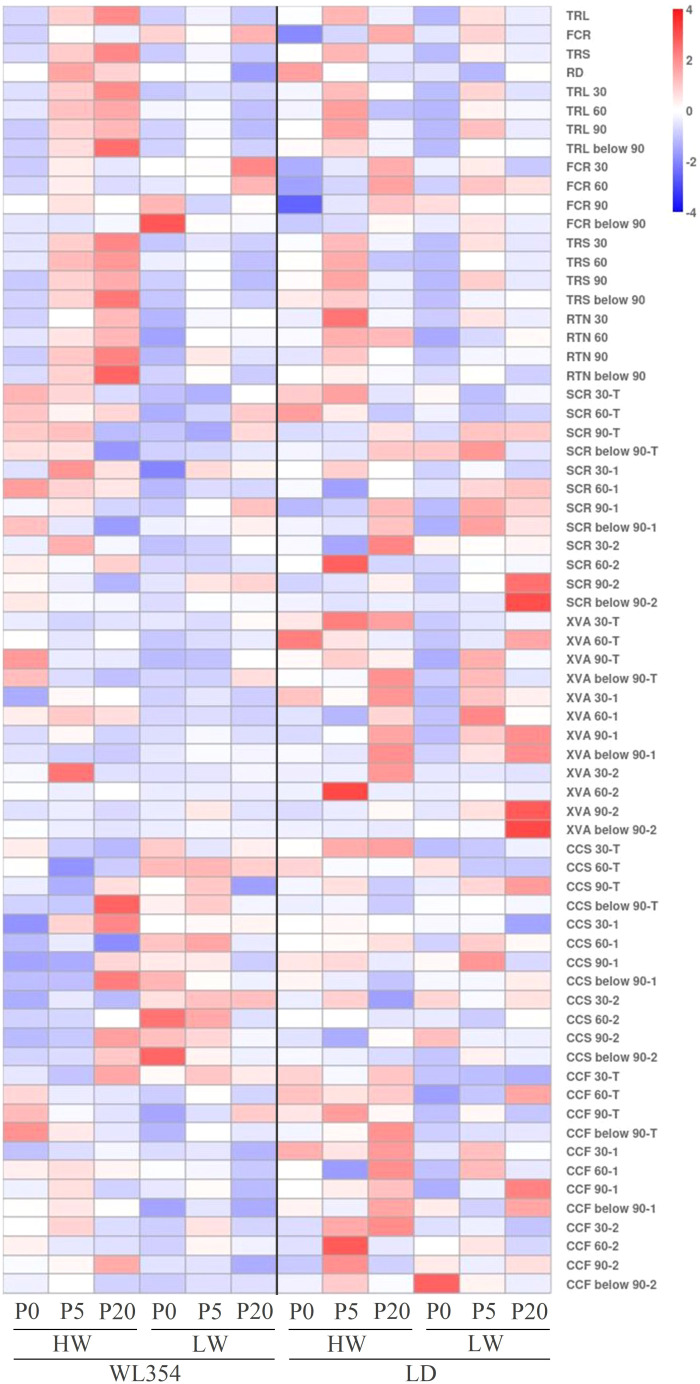
Overall assessment of root traits response characteristics in WL354 and LD alfalfa to phosphorus deficiency under drought stress in the rhizoboxes.

The heat map summarized the overall assessment of the root trait response characteristics of two alfalfa cultivars to P deficiency under drought stress in the rhizoboxes (**Figure 6**). Under the LW and P0 treatment, WL354 increased the proportion of fine roots in the soil layers below 60 cm. It also maintained the xylem vessel area of lateral roots below the 90 cm soil layer and enlarged the cortical cells of taproots in the 0–30 cm soil layer and of 2^nd^ lateral roots in the 30–60 and below 90 cm soil layers. Furthermore, WL354 reduced the cortical cell files in taproots and lateral roots across all soil layers. Under the LW and P0 treatment, LD maintained the cortical cell size of taproots in the 30–60 and below 90 cm soil layers and of 2^nd^ lateral roots in the 0–30 cm soil layer, and increased the cortical cell size of 2^nd^ lateral roots in the 60–90 cm soil layer. Furthermore, LD reduced the cortical cell files in taproots across all soil layers and in 1^st^ lateral roots in soil layers above 90 cm.

### 3.4 Effects of water and P treatments on P uptake and biomass accumulation in the rhizoboxes

**Figure 7:**
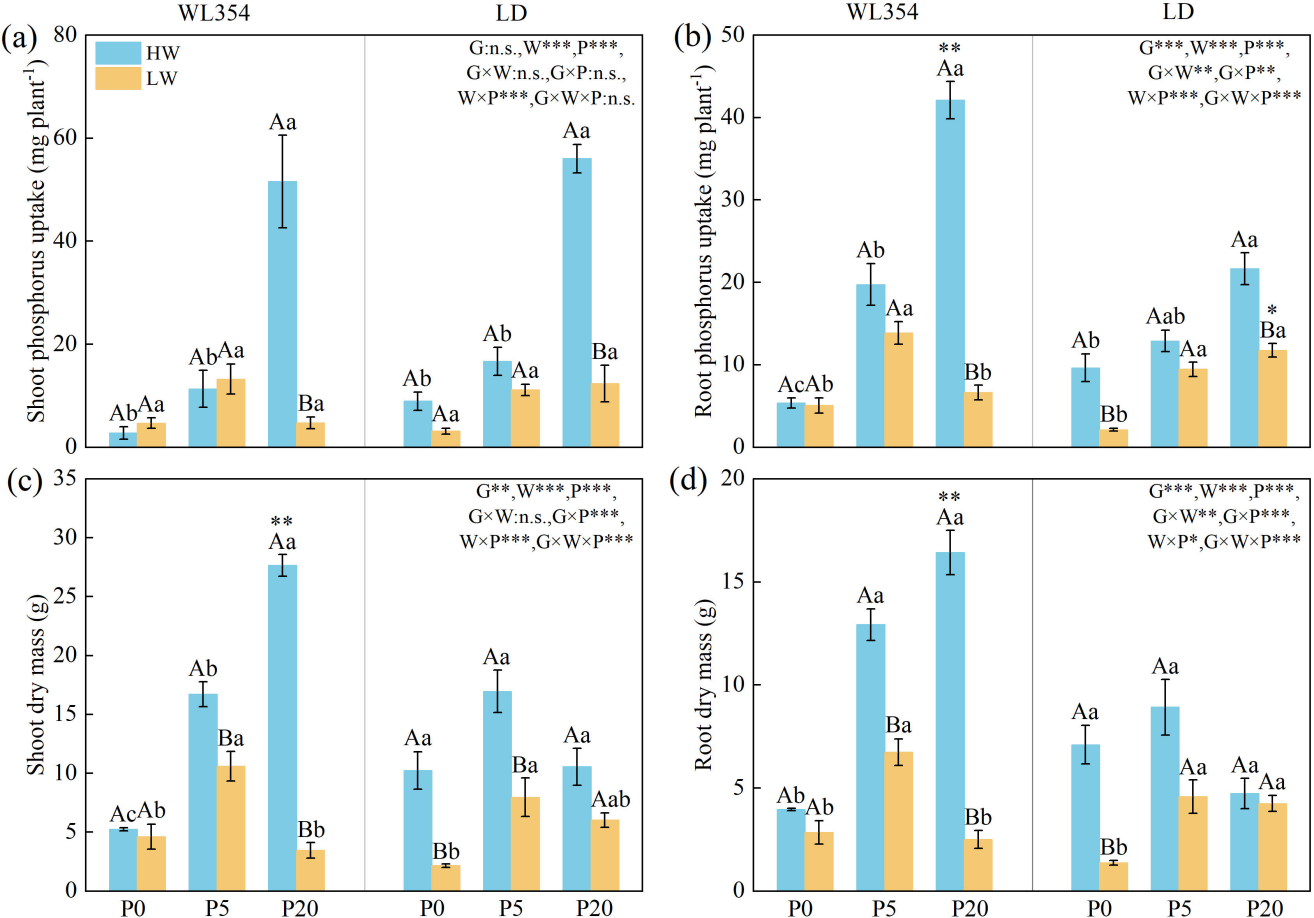
Shoot phosphorus uptake **(a)**, root phosphorus uptake **(b)**, shoot dry mass **(c)**, and root dry mass **(d)** of WL354 and LD alfalfa under different water and phosphorus treatments in the rhizoboxes. Data represented as mean ± SE (n=3). W, water; P, phosphorus; G, genotypes; *P < 0.05; **P < 0.01; ***P < 0.001; n.s., no significant in the three-way ANOVA. When the three-way ANOVA indicated significant main effects of genotype, water, or P, differences within their respective treatment groups were analyzed. Different uppercases indicate significant differences between HW and LW treatments under the same genotypes and phosphorus treatments determined by independent samples t-test (*P* < 0.05); different lowercases indicate significant differences among P0, P5, and P20 treatments under the same genotypes and water treatments according to Tukey’s HSD *post hoc* test (*P* < 0.05); *, ** and *** above the columns indicate significant difference between WL354 and LD under the same water and phosphorus treatments determined by independent samples t-test (*P < 0.05, **P < 0.01, ***P < 0.001). HW, well-watered; LW, drought stress; P0, P5, and P20 refer 0, 5, and 20 mg P kg^-1^ dry soil.

Water, P, and their interaction, significantly influenced the shoot P uptake in two cultivars (**Figure 7a**) (*P* < 0.001). Except for the genotype × water interaction, other factors significantly affected shoot dry mass in both cultivars (**Figure 7c**) (*P* < 0.01). All factors significantly influenced P uptake and root dry mass in both cultivars (**Figures 7b, d**) (*P* < 0.01). Compared to the HW treatment, the LW treatment decreased P uptake as well as shoot and root dry mass for WL354 and LD across most P levels. As P levels increased, the reductions in these traits in WL354 and in P uptake in LD intensified, but the reductions in shoot and root dry mass in LD diminished. Under LW conditions, P uptake and shoot and root dry mass in WL354, as well as shoot and root dry mass in LD, peaked at P5. P uptake in LD increased with increasing P levels.

### 3.5 Correlation analysis of root traits with shoot P uptake in the rhizoboxes

**Figure 8:**
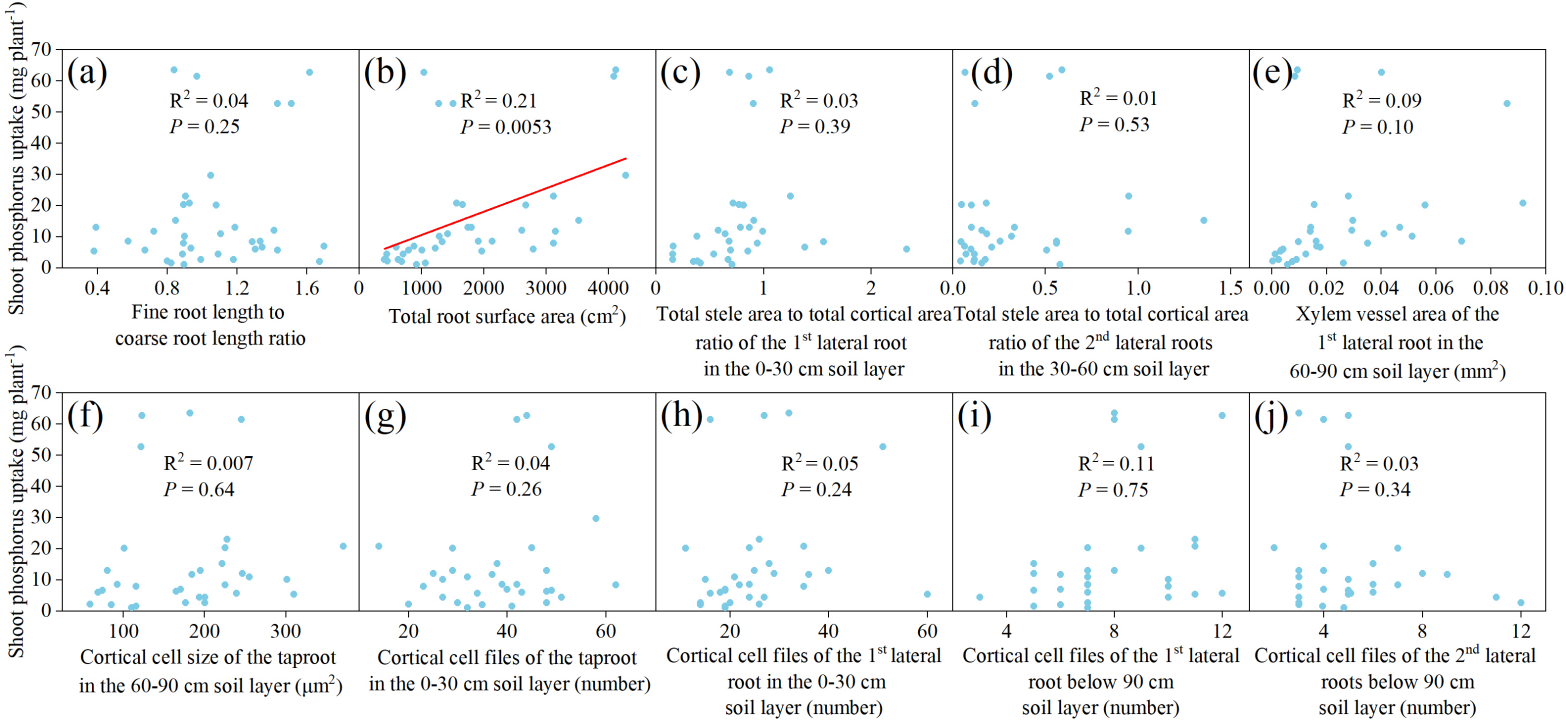
Simple linear correlation between shoot phosphorus uptake and **(a)** fine root length to coarse root length ratio, **(b)** total root surface area, **(c)** total stele area to total cortical area ratio of the 1^st^ lateral root in the 0–30 cm soil layer, **(d)** total stele area to total cortical area ratio of the 2^nd^ lateral roots in the 30–60 cm soil layer, **(e)** xylem vessel area of the 1^st^ lateral root in the 60–90 cm soil layer, **(f)** cortical cell size of the taproot in the 60–90 cm soil layer, **(g)** cortical cell files of the taproot in the 0–30 cm soil layer, **(h)** cortical cell files of the 1^st^ lateral root in the 0–30 cm soil layer, **(i)** cortical cell files of the 1^st^ lateral root below 90 cm soil layer, **(j)** cortical cell files of the 2^nd^ lateral roots below 90 cm soil layer, in the rhizoboxes.

To avoid the impact of multicollinearity among root traits, principal component analysis was performed separately on root morphological traits (excluding dynamic traits) and anatomical traits after standardization. For root morphological traits, 2 principal components (PCs) had eigenvalues greater than 1, with a cumulative contribution ratio of 79.79% (**Supplementary Table 5**). For root anatomical traits, 13 PCs had eigenvalues greater than 1, with a cumulative contribution ratio of 89.03% (**Supplementary Table 6**). The root trait with the largest absolute loading value in each PC was selected for correlation analysis (3 root morphological and 13 root anatomical traits). Pearson correlation analysis showed that some root morphological or anatomical traits were significantly positively correlated, indicating collinearity (**Supplementary Figure 4**). Therefore, the trait with the product of its largest absolute loading and the corresponding PC’s contribution ratio was retained for further analysis. As a result, 1 root morphological trait and 9 anatomical traits were retained, with no significant positive correlations among the 9 anatomical traits. Subsequently, these 10 traits were used for simple linear regression analysis to examine their relationships with shoot P uptake (**Figure 8**). The results showed that only total root surface area was significantly positively correlated with shoot P uptake (R^2^ = 0.21, *P* = 0.0053).

### 3.6 Validating the correlation of root traits with shoot P uptake in the field

**Figure 9:**
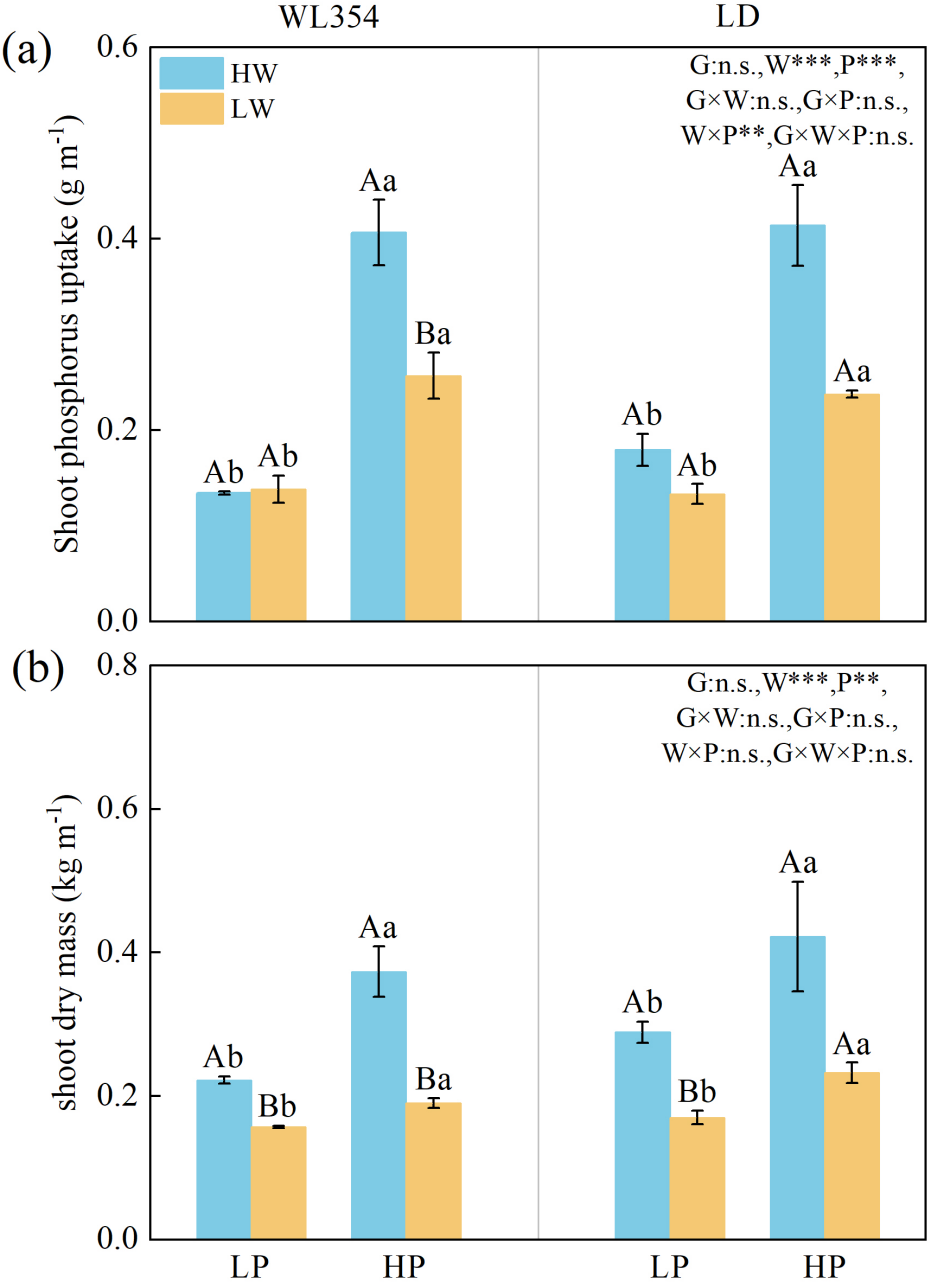
Shoot phosphorus uptake **(a)**, and shoot dry mass **(b)** of WL354 and LD alfalfa under different water and phosphorus treatments in the field. Data represented as mean ± SE (n=3). W, water; P, phosphorus; G, genotypes; *P < 0.05; **P < 0.01; ***P < 0.001; n.s., no significant in the three-way ANOVA. When the three-way ANOVA indicated significant main effects of genotype, water, or P, differences within their respective treatment groups were analyzed. Different uppercases indicate significant differences between HW and LW treatments under the same genotypes and phosphorus treatments determined by independent samples t-test (*P* < 0.05); different lowercases indicate significant differences between LP and HP treatments under the same genotypes and water treatments determined by independent samples t-test (*P* < 0.05); *, ** and *** above the columns indicate significant difference between WL354 and LD under the same water and phosphorus treatments determined by independent samples t-test (*: *P* < 0.05, **: *P* < 0.01, ***: *P* < 0.001). HW, well-watered; LW, drought stress; HP, high P; LP, low P.

Genotype significantly influenced the cortical cell size of taproots, with values in LD being higher than in WL354 under the combined LW and LP treatment (**Supplementary Figure 6**). Water treatments significantly affected total root length, total root surface area, xylem vessel area, cortical cell size of taproots, and total stele area to total cortical area ratio of the lateral roots (**Supplementary Figures 5**, **6**), shoot P uptake, and dry mass (**Figure 9**). These traits were lower under the LW treatment compared to the HW treatment, except for the total stele area to total cortical area ratio of lateral roots. P treatments significantly influenced total root length, xylem vessel area, and cortical cell files of taproots, shoot P uptake and dry mass, with these traits being higher under the HP treatment.

**Figure 10:**
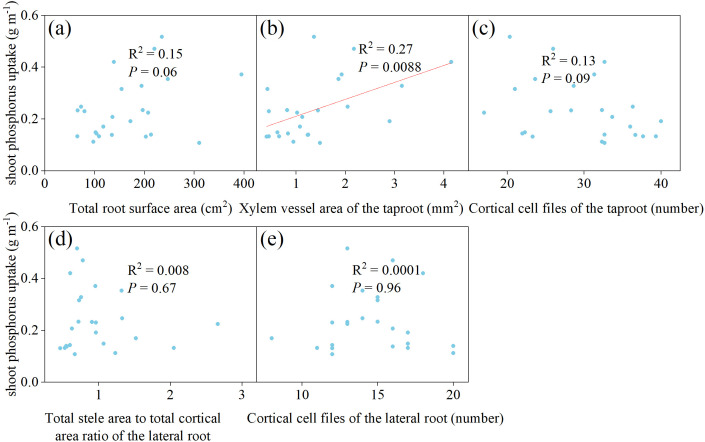
Simple linear correlation between shoot phosphorus uptake and **(a)** total root surface area, **(b)** xylem vessel area of the taproot, **(c)** cortical cell files of the taproot, **(d)** total stele area to total cortical area ratio of the lateral root, **(e)** cortical cell files of the lateral root, in the field.

Subsequently, the relationships between root traits and shoot P uptake were validated. Similarly, to avoid the influence of multicollinearity among root traits, principal component analysis was performed separately on root morphological and anatomical traits after standardization. For root morphological traits, 2 PCs had eigenvalues greater than 1, with a cumulative contribution ratio of 89.52% (**Supplementary Table 7**). For root anatomical traits, 4 PCs had eigenvalues greater than 1, with a cumulative contribution ratio of 80.52% (**Supplementary Table 8**). The root trait with the largest absolute loading value in each PC was selected for correlation analysis (2 root morphological and 4 anatomical traits). Pearson correlation analysis showed that total root length and total root surface area had a significant positive correlation, indicating collinearity (**Supplementary Figure 7**). Given that total root surface area exhibited a high loading on a PC with a substantial contribution rate, it was selected for subsequent analysis. These 5 traits were then used for simple linear regression analysis to examine their relationships with shoot P uptake (**Figure 10**). The results showed that only the xylem vessel area of the taproot was significantly positively correlated with shoot P uptake (R^2^ = 0.21, *P* = 0.0053).

## 4 Discussion

### 4.1 Adaptive strategies of root traits in two alfalfa cultivars to P deficiency under drought stress

Different cultivars of the same species may exhibit distinct root adaptation strategies under abiotic stress (Fan et al., 2015). Fine and coarse roots are the main organs responsible for resource absorption and transportation, respectively (Wang et al., 2017). Fine roots play a key role in the carbon economy at both the plant and ecosystem levels, as approximately 40% of the net carbon fixed by photosynthesis is allocated to fine roots (Jones et al., 2009). This carbon is used for maintaining existing fine roots and constructing new roots to facilitate resource acquisition (Roumet et al., 2016). An increased proportion of fine roots is recognized as a critical adaptive strategy, as fine roots possess a higher surface area-to-volume ratio, which facilitates greater soil contact and improves the uptake efficiency of water, P, and other essential nutrients (Wang et al., 2020). In this study, the increased fine root length in deep soil layers observed in WL354 may help improve nitrate foraging and water acquisition, thereby enabling the plant to maintain physiological activities and growth under low-P and drought stress. This is crucial given that water and nitrate are distributed in deeper soil layers compared to P (Lynch, 2013). These findings highlight the importance of root architectural adaptation in mediating plant resilience under nutrient and water scarcity.

Root anatomy is important for the acquisition and transportation of nutrients and water within plants, and it primarily determines the metabolic costs of root construction and maintenance (Burton et al., 2012). The number and diameter of xylem vessels reflect the ability of plants to transport nutrients and water vertically (Zhao et al., 2023). Under drought and low P conditions, WL354 maintained the xylem vessel area of lateral roots in deep soil layers relatively stable. This enabled it to maintain its capacity to vertically transport soil moisture and nutrients, thus providing crucial support for its growth. Larger cortical cell size and fewer cortical cell files reduce the radial transport resistance of nutrients and water in the root system and decrease the metabolic costs of soil exploration, effectively creating “cheaper” roots that facilitate resource acquisition under drought conditions (Chimungu et al., 2014; De Bauw et al., 2019; Lynch, 2013). Under low P and drought conditions, WL354 enlarged the cortical cells of taproots and 2^nd^ lateral roots, and simultaneously reduced the cortical cell files in taproots and lateral roots across all soil layers. This adaptation helped reduce radial transport resistance of water and nutrients. In contrast, LD mainly maintained the cortical cell size of taproots and 2^nd^ lateral roots, and reduced the cortical cell files in taproots and in 1^st^ lateral roots across all soil layers to reduce radial transport resistance. In maize (*Zea mays* L.), roots reduce metabolic costs by decreasing the number of cortical aerenchyma and increasing cortical cell size, which improves N and P absorption and utilization of roots, synergistically increasing grain yield and nutrients utilization efficiency of the whole plant (Chen et al., 2022).

### 4.2 The effect of root trait changes on shoot P uptake in two alfalfa cultivars

The root system is the primary organ responsible for absorbing water and nutrients, anchoring the plant, and influencing both yield and quality (Lu et al., 2019). Our results revealed distinct correlations between root traits and shoot P uptake in both the rhizobox and field experiments. In the rhizobox experiment, shoot P uptake was significantly and positively correlated with total root surface area. Since phosphate ions have low mobility and diffusion coefficients in soil, P acquisition is primarily limited by the volume of soil explored by the root system (Lynch, 2011). Total root surface area represents the primary absorptive interface between the plant and the soil, directly determining the capacity for water uptake and the interception of immobile nutrients (Lynch, 2022). In the confined space of rhizoboxes, maximizing the root-soil contact interface through increased surface area is the most direct strategy to minimize the diffusion path length and enhance P uptake (McLachlan et al., 2020). This suggests that in confined spaces, morphological expansion, such as total root surface area, is the primary driver of P uptake.

In the field experiment, the significant correlation between the xylem vessel area of the taproot and shoot P uptake suggested that hydraulic conductance becomes a limiting factor for nutrient acquisition under natural, variable environments. Although root sampling was confined to the 0–30 cm soil layer, the taproot in this section serves as the central hydraulic conduit connecting the entire root system to the shoot, making efficient water transport through this primary pathway critical (Lynch, 2013). Larger xylem vessels in the taproot in the topsoil improve nutrient and water transport vertically (Zhao et al., 2023), thereby facilitating water transport to sustain transpiration and nutrient movement (Chimungu et al., 2014). Although P moves primarily by diffusion, its uptake is physiologically coupled with plant water status. Sufficient water uptake is required to maintain the concentration gradient at the root surface and support the metabolic costs of ion absorption (Mimura and Reid, 2024). Therefore, under field conditions that may be prone to water deficits, ensuring efficient transport through the xylem vessel area of the taproot appears to be more critical for sustaining shoot P uptake than surface area alone.

### 4.3 Implications for breeding and management, and study limitations

Synthesizing the root strategies adopted by WL354 and LD, we concluded that an increased proportion of fine roots and xylem vessel area of lateral roots in deeper soil layers, together with enlarged cortical cells and fewer cortical cell files in both taproots and lateral roots across all soil layers, contribute to alfalfa’s adaptation to drought and low P stress. Furthermore, total root surface area and xylem vessel area of the taproot were significantly positively correlated with shoot P uptake. Our findings suggest that these root traits could serve as potential candidate targets for breeding alfalfa cultivars with enhanced tolerance to drought and low P availability, potentially contributing to improved yield in arid areas with alkaline, low P soils.

In semiarid environments, P application can increase the yield of alfalfa (Fan et al., 2016). However, when P application exceeds the growth and development needs of alfalfa, issues such as reduced yield, low fertilizer use efficiency, and environmental pollution arise (Zhang et al., 2020). Therefore, proper fertilizer application can increase crop yield and reduce environmental pollution. In this rhizobox experiment, applying 5 mg P kg^-1^ dry soil maximized biomass relative to 20 mg P kg^-1^ dry soil under drought conditions for both WL354 and LD.

This study revealed the morphological and anatomical strategies of alfalfa roots in response to drought and low P stress. However, the effects of arbuscular mycorrhizal fungi and root exudates on soil P uptake were not considered. In addition, only root traits within the top 30 cm of soil were measured in the field experiment. Future studies should collect root samples from all soil layers and integrate root morphological, anatomical traits, arbuscular mycorrhizal fungi and root exudates to comprehensively investigate alfalfa’s adaptive strategies to drought and low P stress.

## 5 Conclusions

To cope with drought and low P stress, WL354 adopted a resource acquisition strategy by increasing fine roots in deep soil layers, maintaining xylem vessel area, enlarging cortical cells, and reducing cortical cell files. In contrast, LD displayed a more conservative anatomical response, primarily maintaining cortical cell size and reducing cortical cell files. Shoot P uptake was significantly positively correlated with both total root surface area and taproot xylem vessel area. These findings highlight the value of integrating morphological and anatomical traits as novel alfalfa breeding targets for drought and low P stress tolerance, and validate the application of 5 mg P kg^-1^ dry soil as an effective management practice under our experimental conditions, which offers potential implications for field fertilization strategies in arid areas with alkaline, low P soils. However, as field sampling was limited to the topsoil, future research must integrate deep-soil sampling along with rhizosphere interactions to fully unravel the adaptive mechanisms of alfalfa roots to drought and low P stress.

## Data Availability

The raw data supporting the conclusions of this article will be made available by the authors, without undue reservation.
