# Supplementary material for: Response of root morphology and anatomy of two alfalfa cultivars with contrasting root system architecture to phosphorus deficiency under drought stress
Source: Front Plant Sci. 2026 Mar 24;17:1741660. doi: 10.3389/fpls.2026.1741660 (PMC13055590; doi:10.3389/fpls.2026.1741660)
Supplement: Supplementary file 1 [file SupplementaryFile1.docx]

***Supplementary Material***
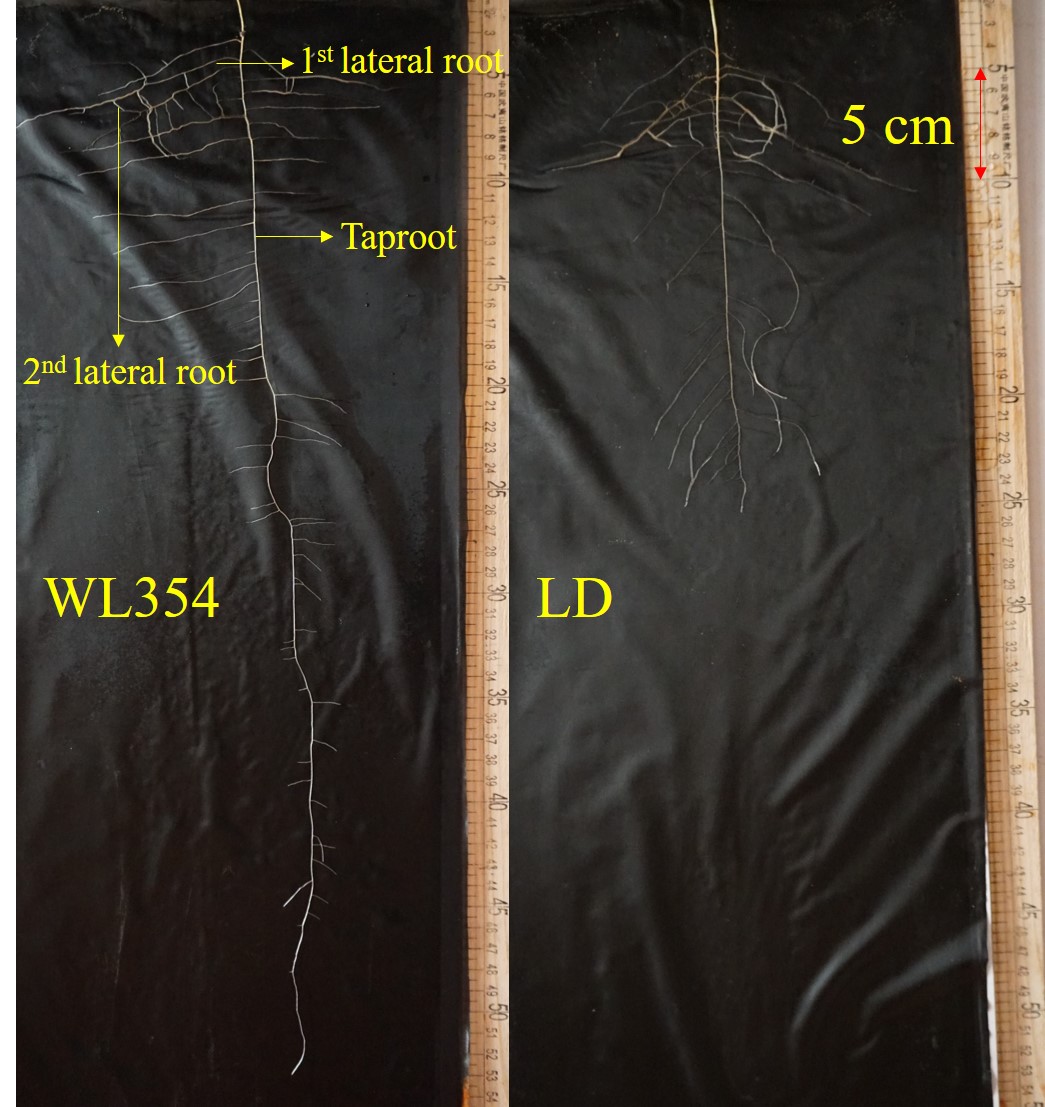


**Supplementary Figure 1.** Root system comparison of WL354 and LD grow in the rhizoboxes under normal growing conditions at the seedling stage. Bar = 5 cm.


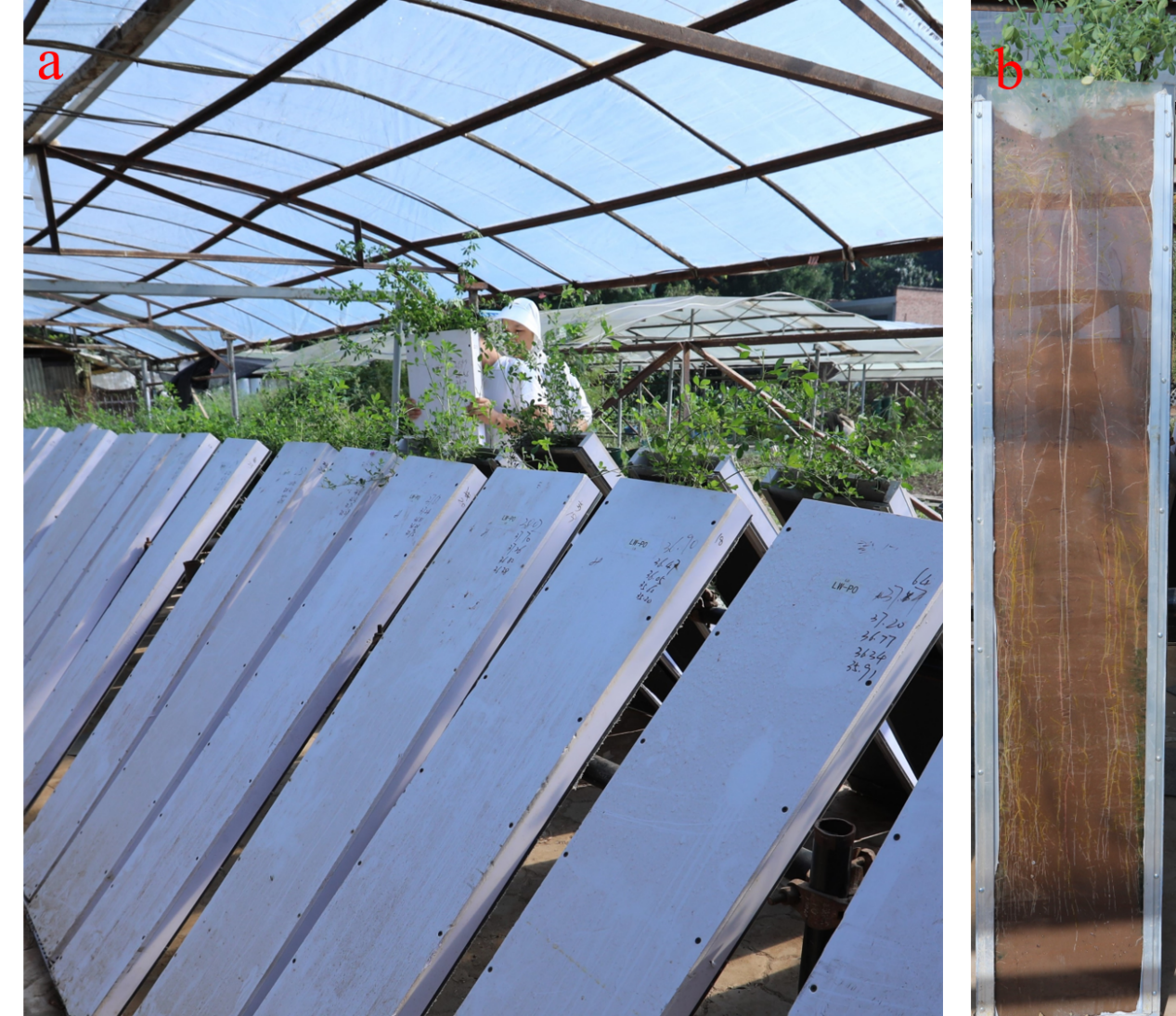


**Supplementary Figure 2.** The growth condition of alfalfa (a) and its roots (b) in the rhizoboxes.


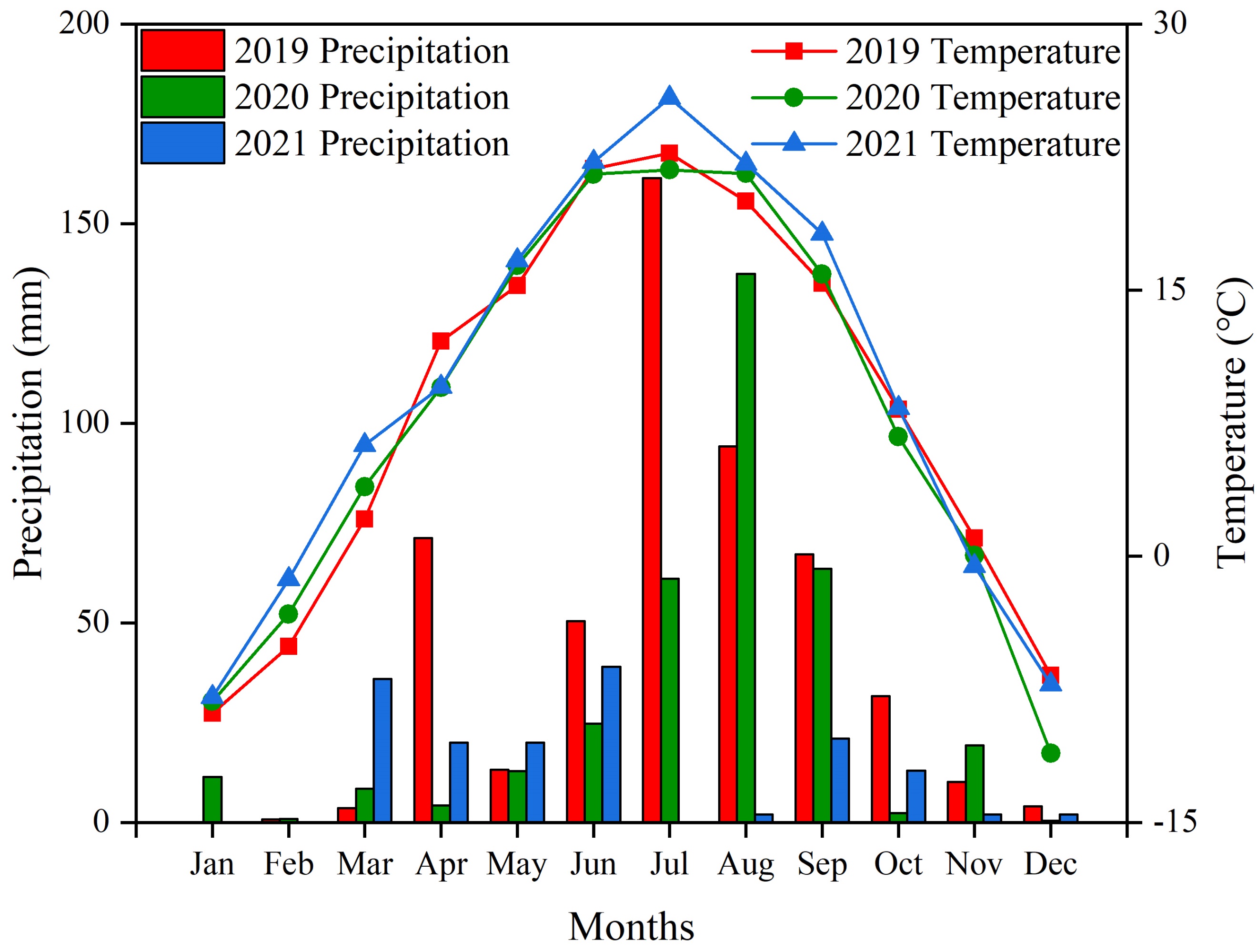
 **Supplementary Figure 3.** Monthly average values of precipitation and air temperature at the experimental site during 2019, 2020 and 2021.

**Supplementary Table 1.** Abbreviations of the traits used in the rhizoboxes.

| Traits | Abbreviations |
| --- | --- |
| Total root length | TRL |
| Fine root length to coarse root length ratio | FCR |
| Total root surface area | TRS |
| Root diameter | RD |
| Total root length in the 0-30 cm soil layer | TRL 30 |
| Total root length in the 30-60 cm soil layer | TRL 60 |
| Total root length in the 60-90 cm soil layer | TRL 90 |
| Total root length below 90 cm soil layer | TRL below 90 |
| Fine root length to coarse root length ratio in the 0-30 cm soil layer | FCR 30 |
| Fine root length to coarse root length ratio in the 30-60 cm soil layer | FCR 60 |
| Fine root length to coarse root length ratio in the 60-90 cm soil layer | FCR 90 |
| Fine root length to coarse root length ratio below 90 cm soil layer | FCR below 90 |
| Total root surface area in the 0-30 cm soil layer | TRS 30 |
| Total root surface area in the 30-60 cm soil layer | TRS 60 |
| Total root surface area in the 60-90 cm soil layer | TRS 90 |
| Total root surface area below 90 cm soil layer | TRS below 90 |
| Number of root tips in the 0-30 cm soil layer | RTN 30 |
| Number of root tips in the 30-60 cm soil layer | RTN 60 |
| Number of root tips in the 60-90 cm soil layer | RTN 90 |
| Number of root tips below 90 cm soil layer | RTN below 90 |
| Total stele area to total cortical area ratio of the taproot in the 0-30 cm soil layer | SCR 30-T |
| Total stele area to total cortical area ratio of the taproot in the 30-60 cm soil layer | SCR 60-T |
| Total stele area to total cortical area ratio of the taproot in the 60-90 cm soil layer | SCR 90-T |
| Total stele area to total cortical area ratio of the taproot below 90 cm soil layer | SCR below 90-T |
| Total stele area to total cortical area ratio of the 1^st^ lateral root in the 0-30 cm soil layer | SCR 30-1 |
| Total stele area to total cortical area ratio of the 1^st^ lateral root in the 30-60 cm soil layer | SCR 60-1 |
| Total stele area to total cortical area ratio of the 1^st^ lateral root in the 60-90 cm soil layer | SCR 90-1 |
| Total stele area to total cortical area ratio of the 1^st^ lateral root below 90 cm soil layer | SCR below 90-1 |
| Total stele area to total cortical area ratio of the 2^nd^ lateral roots in the 0-30 cm soil layer | SCR 30-2 |
| Total stele area to total cortical area ratio of the 2^nd^ lateral roots in the 30-60 cm soil layer | SCR 60-2 |
| Total stele area to total cortical area ratio of the 2^nd^ lateral roots in the 60-90 cm soil layer | SCR 90-2 |
| Total stele area to total cortical area ratio of the 2^nd^ lateral roots below 90 cm soil layer | SCR below 90-2 |
| Xylem vessel area of the taproot in the 0-30 cm soil layer | XVA 30-T |
| Xylem vessel area of the taproot in the 30-60 cm soil layer | XVA 60-T |
| Xylem vessel area of the taproot in the 60-90 cm soil layer | XVA 90-T |
| Xylem vessel area of the taproot below 90 cm soil layer | XVA below 90-T |
| Xylem vessel area of the 1^st^ lateral root in the 0-30 cm soil layer | XVA 30-1 |
| Xylem vessel area of the 1^st^ lateral root in the 30-60 cm soil layer | XVA 60-1 |
| Xylem vessel area of the 1^st^ lateral root in the 60-90 cm soil layer | XVA 90-1 |
| Xylem vessel area of the 1^st^ lateral root below 90 cm soil layer | XVA below 90-1 |
| Xylem vessel area of the 2^nd^ lateral roots in the 0-30 cm soil layer | XVA 30-2 |
| Xylem vessel area of the 2^nd^ lateral roots in the 30-60 cm soil layer | XVA 60-2 |
| Xylem vessel area of the 2^nd^ lateral roots in the 60-90 cm soil layer | XVA 90-2 |
| Xylem vessel area of the 2^nd^ lateral roots below 90 cm soil layer | XVA below 90-2 |
| Cortical cell size of the taproot in the 0-30 cm soil layer | CCS 30-T |
| Cortical cell size of the taproot in the 30-60 cm soil layer | CCS 60-T |
| Cortical cell size of the taproot in the 60-90 cm soil layer | CCS 90-T |
| Cortical cell size of the taproot below 90 cm soil layer | CCS below 90-T |
| Cortical cell size of the 1^st^ lateral root in the 0-30 cm soil layer | CCS 30-1 |
| Cortical cell size of the 1^st^ lateral root in the 30-60 cm soil layer | CCS 60-1 |
| Cortical cell size of the 1^st^ lateral root in the 60-90 cm soil layer | CCS 90-1 |
| Cortical cell size of the 1^st^ lateral root below 90 cm soil layer | CCS below 90-1 |
| Cortical cell size of the 2^nd^ lateral roots in the 0-30 cm soil layer | CCS 30-2 |
| Cortical cell size of the 2^nd^ lateral roots in the 30-60 cm soil layer | CCS 60-2 |
| Cortical cell size of the 2^nd^ lateral roots in the 60-90 cm soil layer | CCS 90-2 |
| Cortical cell size of the 2^nd^ lateral roots below 90 cm soil layer | CCS below 90-2 |
| Cortical cell files of the taproot in the 0-30 cm soil layer | CCF 30-T |
| Cortical cell files of the taproot in the 30-60 cm soil layer | CCF 60-T |
| Cortical cell files of the taproot in the 60-90 cm soil layer | CCF 90-T |
| Cortical cell files of the taproot below 90 cm soil layer | CCF below 90-T |
| Cortical cell files of the 1^st^ lateral root in the 0-30 cm soil layer | CCF 30-1 |
| Cortical cell files of the 1^st^ lateral root in the 30-60 cm soil layer | CCF 60-1 |
| Cortical cell files of the 1^st^ lateral root in the 60-90 cm soil layer | CCF 90-1 |
| Cortical cell files of the 1^st^ lateral root below 90 cm soil layer | CCF below 90-1 |
| Cortical cell files of the 2^nd^ lateral roots in the 0-30 cm soil layer | CCF 30-2 |
| Cortical cell files of the 2^nd^ lateral roots in the 30-60 cm soil layer | CCF 60-2 |
| Cortical cell files of the 2^nd^ lateral roots in the 60-90 cm soil layer | CCF 90-2 |
| Cortical cell files of the 2^nd^ lateral roots below 90 cm soil layer | CCF below 90-2 |
| Shoot phosphorus uptake | SPU |

**Supplementary Table 2.** Three-way ANOVA of root morphological traits in each soil layer under different water and phosphorus treatments in the rhizoboxes. W: water, P: phosphorus, G: genotypes. *P* values < 0.05 are bolded.

| Traits | G | W | P | G×W | G×P | W×P | G×W×P |
| --- | --- | --- | --- | --- | --- | --- | --- |
| TRL 30 | 0.73 | **0.00** | **0.00** | **0.00** | **0.00** | **0.00** | **0.00** |
| TRL 60 | **0.01** | **0.00** | **0.00** | **0.00** | **0.00** | **0.00** | **0.00** |
| TRL 90 | 0.11 | **0.00** | **0.00** | 0.18 | **0.01** | 0.22 | **0.00** |
| TRL below 90 | **0.00** | **0.00** | **0.00** | **0.00** | **0.00** | **0.00** | **0.00** |
| FCR 30 | 0.05 | **0.04** | **0.04** | **0.01** | 0.84 | 0.18 | **0.00** |
| FCR 60 | 0.77 | **0.01** | **0.01** | 0.75 | **0.02** | 0.83 | **0.00** |
| FCR 90 | 0.12 | 0.12 | 0.12 | 0.11 | **0.01** | **0.00** | 0.05 |
| FCR below 90 | **0.00** | **0.00** | **0.00** | **0.00** | **0.00** | **0.00** | **0.00** |
| TRS 30 | 0.29 | **0.00** | **0.00** | **0.00** | **0.00** | **0.00** | **0.00** |
| TRS 60 | **0.00** | **0.00** | **0.00** | **0.00** | **0.00** | **0.01** | **0.00** |
| TRS 90 | 0.06 | **0.00** | **0.00** | 0.06 | **0.00** | 0.05 | **0.00** |
| TRS below 90 | **0.00** | **0.00** | **0.00** | **0.00** | **0.00** | **0.00** | **0.00** |
| RTN 30 | 0.14 | **0.00** | **0.00** | 0.77 | **0.00** | 0.62 | 0.07 |
| RTN 60 | 0.65 | **0.00** | **0.00** | 0.59 | 1.00 | 0.98 | 0.61 |
| RTN 90 | 0.18 | **0.00** | **0.00** | 0.26 | 0.13 | 0.27 | **0.02** |
| RTN below 90 | 0.31 | **0.02** | **0.02** | 0.25 | 0.21 | 0.17 | 0.24 |

**Supplementary Table 3.** Three-way ANOVA of dynamic changes in root morphological traits under different water and phosphorus treatments in the rhizoboxes. W: water, P: phosphorus, G: genotypes. *P* values < 0.05 are bolded.

| Traits | G | W | P | G×W | G×P | W×P | G×W×P |
| --- | --- | --- | --- | --- | --- | --- | --- |
| TRL 9d | 0.08 | 0.34 | **0.01** | 0.06 | 0.11 | 0.83 | 0.23 |
| TRL 15d | 0.30 | 0.41 | **0.03** | 0.09 | 0.36 | 0.91 | 0.17 |
| TRL 30d | 0.12 | **0.00** | **0.00** | **0.01** | 0.23 | 0.52 | 0.08 |
| TRL 50d | 0.49 | **0.00** | **0.00** | 0.11 | 0.33 | 0.75 | 0.13 |
| RTN 9d | 0.11 | 0.08 | **0.04** | 0.33 | **0.04** | 0.68 | 0.08 |
| RTN 15d | 0.15 | 0.05 | **0.04** | 0.38 | 0.09 | 0.67 | 0.08 |
| RTN 30d | **0.02** | **0.00** | **0.00** | **0.03** | **0.02** | 0.09 | **0.02** |
| RTN 50d | 0.71 | **0.00** | **0.00** | 0.34 | 0.08 | 0.25 | 0.07 |
| MRD 9d | 0.30 | 0.79 | 0.33 | 0.11 | 0.28 | 0.99 | 0.20 |
| MRD 15d | 0.50 | 0.80 | 0.25 | 0.18 | 0.37 | 0.93 | 0.23 |
| MRD 30d | 0.87 | 0.83 | 0.19 | 0.08 | 0.27 | 0.51 | 0.14 |
| MRD 50d | 0.82 | 0.40 | **0.03** | **0.01** | 0.38 | 0.15 | 0.24 |

**Supplementary Table 4.** Three-way ANOVA of root anatomical traits in each soil layer under different water and phosphorus treatments in the rhizoboxes. W: water, P: phosphorus, G: genotypes. *P* values < 0.05 are bolded.

| Traits | G | W | P | G×W | G×P | W×P | G×W×P |
| --- | --- | --- | --- | --- | --- | --- | --- |
| SCR 30-T | 0.88 | **0.03** | 0.77 | 0.79 | 1.00 | 0.07 | 0.61 |
| SCR 60-T | 0.29 | 0.10 | 0.67 | 0.99 | 0.42 | 0.30 | 0.64 |
| SCR 90-T | 0.41 | 0.97 | 0.08 | **0.01** | 0.06 | 0.10 | 0.08 |
| SCR below 90-T | 0.38 | 0.95 | 0.83 | 0.60 | 0.92 | 0.97 | 0.38 |
| SCR 30-1 | 0.64 | 0.18 | 0.07 | 0.84 | 0.27 | 0.90 | 0.80 |
| SCR 60-1 | 0.84 | 0.31 | 0.45 | **0.00** | 0.70 | 0.13 | 0.70 |
| SCR 90-1 | 0.68 | 0.35 | **0.02** | 0.62 | 0.43 | 0.41 | 0.06 |
| SCR below 90-1 | 0.59 | 0.52 | 0.47 | 0.84 | **0.02** | 0.24 | 0.21 |
| SCR 30-2 | 0.34 | 0.51 | 0.49 | 0.43 | 0.07 | 0.95 | 0.06 |
| SCR 60-2 | 0.22 | **0.00** | 0.06 | 0.59 | **0.00** | **0.02** | **0.00** |
| SCR 90-2 | 0.65 | 0.12 | 0.37 | 0.74 | 0.31 | 0.28 | 0.96 |
| SCR below 90-2 | **0.02** | **0.00** | **0.00** | **0.00** | **0.00** | **0.00** | **0.00** |
| XVA 30-T | 0.05 | **0.00** | 0.44 | **0.00** | 0.25 | 0.43 | 0.29 |
| XVA 60-T | 0.68 | 0.79 | 0.37 | 0.53 | 0.48 | 0.29 | 0.50 |
| XVA 90-T | 0.22 | **0.01** | **0.01** | **0.01** | **0.00** | **0.01** | 0.10 |
| XVA below 90-T | 0.42 | 0.64 | 0.80 | 0.84 | 0.32 | 0.27 | 0.14 |
| XVA 30-1 | 0.09 | 0.81 | 0.14 | 0.65 | 0.40 | 0.90 | 0.50 |
| XVA 60-1 | 0.98 | 0.32 | 0.10 | **0.00** | 0.53 | 0.09 | 0.08 |
| XVA 90-1 | **0.02** | 0.88 | **0.01** | 0.71 | **0.01** | 0.74 | 0.46 |
| XVA below 90-1 | **0.04** | 0.35 | 0.07 | 0.87 | **0.04** | 0.70 | 0.88 |
| XVA 30-2 | 0.84 | 0.16 | 0.68 | 0.90 | 0.32 | 0.62 | 0.32 |
| XVA 60-2 | **0.00** | **0.00** | **0.00** | **0.00** | **0.00** | **0.00** | **0.00** |
| XVA 90-2 | 0.09 | **0.04** | 0.20 | 0.20 | 0.15 | 0.48 | 0.56 |
| XVA below 90-2 | **0.00** | **0.00** | **0.00** | **0.00** | **0.00** | **0.00** | **0.00** |
| CCS 30-T | 0.90 | 0.21 | 0.90 | **0.01** | 0.23 | 0.87 | 0.58 |
| CCS 60-T | 0.77 | 0.37 | 0.53 | 0.13 | 0.84 | 0.90 | 0.53 |
| CCS 90-T | 0.05 | **0.00** | 0.06 | **0.00** | **0.03** | 0.29 | **0.00** |
| CCS below 90-T | **0.00** | **0.04** | 0.09 | 0.58 | **0.00** | **0.00** | **0.00** |
| CCS 30-1 | 0.87 | 0.77 | 0.20 | 0.08 | 0.67 | 0.51 | 0.36 |
| CCS 60-1 | 0.17 | 0.11 | 0.29 | 0.16 | 0.26 | 0.95 | 0.69 |
| CCS 90-1 | **0.01** | 0.06 | 0.06 | 0.18 | 0.05 | **0.03** | 0.15 |
| CCS below 90-1 | 0.16 | 0.30 | **0.02** | 0.77 | **0.02** | 0.05 | **0.00** |
| CCS 30-2 | 0.56 | **0.01** | 0.37 | **0.02** | 0.34 | 0.06 | 0.28 |
| CCS 60-2 | **0.03** | **0.01** | 0.10 | **0.00** | 0.14 | 0.05 | **0.00** |
| CCS 90-2 | 0.67 | 0.09 | 0.99 | 0.16 | 0.43 | 0.80 | 0.73 |
| CCS below 90-2 | **0.01** | 0.10 | 0.60 | 0.11 | **0.03** | **0.03** | **0.00** |
| CCF 30-T | 0.32 | 0.30 | 0.66 | **0.02** | 0.41 | 0.32 | 0.70 |
| CCF 60-T | 0.81 | 0.25 | 0.23 | 0.70 | **0.01** | 0.20 | 0.43 |
| CCF 90-T | 0.61 | **0.01** | **0.03** | 0.50 | **0.01** | **0.02** | 0.39 |
| CCF below 90-T | 0.78 | 0.08 | 0.68 | 0.57 | 0.56 | 0.60 | 0.61 |
| CCF 30-1 | **0.01** | 0.59 | 0.25 | 0.33 | 0.31 | 0.60 | 0.63 |
| CCF 60-1 | 0.71 | 0.11 | 0.55 | 0.40 | 0.27 | **0.04** | 0.08 |
| CCF 90-1 | 0.06 | 0.47 | 0.09 | 0.89 | **0.00** | 0.42 | 0.39 |
| CCF below 90-1 | 0.21 | 0.36 | 0.22 | 0.06 | **0.03** | 0.89 | 0.88 |
| CCF 30-2 | 0.32 | **0.02** | 0.05 | 0.36 | 0.70 | 0.29 | 0.23 |
| CCF 60-2 | 0.09 | 0.05 | **0.00** | 0.12 | **0.00** | 0.40 | **0.00** |
| CCF 90-2 | 0.31 | **0.03** | 0.11 | **0.00** | 0.06 | **0.04** | **0.00** |
| CCF below 90-2 | **0.00** | 0.57 | 0.19 | 0.09 | 0.19 | **0.04** | **0.03** |

**Supplementary Table 5.** Principal component (PC) analysis of root morphological traits (excluding dynamic traits) in the rhizoboxes. The loading with the largest absolute value in each PC is bolded. PCs with eigenvalues > 1 are presented.

| Traits | PC1 | PC2 | PC3 |
| --- | --- | --- | --- |
| TRL | 0.97 | 0.11 | 0.08 |
| FCR | -0.27 | **0.93** | 0.12 |
| TRS | **0.97** | 0.01 | 0.06 |
| RD | 0.33 | -0.69 | 0.21 |
| TRL 30 | 0.92 | 0.17 | 0.04 |
| TRL 60 | 0.91 | 0.01 | 0.24 |
| TRL 90 | 0.92 | 0.18 | 0.03 |
| TRL below 90 | 0.92 | 0.00 | 0.07 |
| FCR 30 | -0.22 | 0.86 | -0.16 |
| FCR 60 | -0.21 | 0.83 | -0.27 |
| FCR 90 | -0.21 | 0.64 | 0.45 |
| FCR below 90 | -0.34 | 0.35 | **0.75** |
| TRS 30 | 0.92 | 0.09 | 0.05 |
| TRS 60 | 0.88 | -0.02 | 0.19 |
| TRS 90 | 0.91 | 0.14 | 0.01 |
| TRS below 90 | 0.89 | -0.06 | 0.02 |
| RTN 30 | 0.76 | 0.20 | -0.24 |
| RTN 60 | 0.63 | 0.24 | -0.45 |
| RTN 90 | 0.83 | 0.21 | -0.15 |
| RTN below 90 | 0.71 | 0.03 | 0.14 |
| Eigenvalue | 11.09 | 3.54 | 1.33 |
| Contributive ratio (%) | 55.47 | 17.68 | 6.63 |
| Cumulative contributive ratio (%) | 55.47 | 73.15 | 79.79 |

**Supplementary Table 6.** Principal component (PC) analysis of root anatomical traits in the rhizoboxes. The loading with the largest absolute value in each PC is bolded. PCs with eigenvalues > 1 are presented.

| Traits | PC1 | PC2 | PC3 | PC4 | PC5 | PC6 | PC7 | PC8 | PC9 | PC10 | PC11 | PC12 | PC13 |
| --- | --- | --- | --- | --- | --- | --- | --- | --- | --- | --- | --- | --- | --- |
| SCR 30-T | 0.18 | 0.59 | 0.27 | -0.26 | -0.10 | 0.20 | 0.16 | -0.10 | 0.30 | -0.07 | 0.11 | 0.05 | **-0.35** |
| SCR 60-T | 0.14 | 0.48 | -0.07 | -0.50 | 0.15 | 0.24 | 0.24 | 0.13 | 0.28 | 0.25 | 0.08 | -0.06 | 0.21 |
| SCR 90-T | 0.72 | 0.13 | -0.06 | -0.21 | 0.10 | -0.07 | -0.02 | 0.21 | 0.33 | 0.00 | -0.05 | -0.23 | -0.20 |
| SCR below 90-T | 0.43 | -0.04 | -0.47 | -0.05 | 0.26 | -0.06 | -0.14 | 0.52 | -0.07 | -0.08 | -0.01 | -0.03 | -0.17 |
| SCR 30-1 | 0.03 | 0.26 | -0.08 | -0.05 | -0.39 | **-0.58** | -0.21 | -0.22 | -0.01 | 0.17 | 0.03 | **0.40** | -0.10 |
| SCR 60-1 | 0.69 | -0.16 | -0.01 | -0.27 | 0.23 | -0.25 | 0.40 | -0.14 | 0.10 | -0.04 | -0.03 | 0.06 | 0.18 |
| SCR 90-1 | 0.66 | -0.13 | -0.22 | 0.06 | -0.08 | -0.01 | -0.21 | 0.16 | 0.20 | 0.15 | -0.41 | 0.10 | -0.16 |
| SCR below 90-1 | 0.50 | -0.13 | -0.24 | 0.12 | 0.48 | -0.20 | -0.36 | -0.15 | 0.30 | 0.14 | -0.01 | 0.03 | 0.05 |
| SCR 30-2 | 0.63 | 0.02 | -0.33 | 0.04 | -0.50 | 0.21 | 0.18 | 0.25 | -0.08 | -0.03 | -0.02 | -0.02 | 0.10 |
| SCR 60-2 | 0.03 | **0.70** | 0.58 | 0.20 | 0.12 | -0.10 | 0.14 | 0.01 | -0.04 | -0.03 | 0.07 | -0.10 | -0.19 |
| SCR 90-2 | 0.31 | -0.37 | 0.50 | -0.03 | -0.14 | 0.02 | -0.37 | -0.30 | -0.07 | -0.11 | -0.37 | 0.06 | 0.26 |
| SCR below 90-2 | 0.48 | -0.45 | 0.55 | -0.28 | -0.11 | -0.04 | 0.01 | -0.09 | 0.14 | 0.15 | 0.24 | -0.10 | -0.08 |
| XVA 30-T | 0.02 | 0.62 | 0.20 | 0.41 | 0.08 | 0.19 | -0.19 | -0.10 | -0.23 | 0.33 | 0.06 | 0.05 | -0.04 |
| XVA 60-T | 0.34 | 0.00 | 0.39 | -0.44 | 0.05 | 0.40 | 0.00 | 0.24 | 0.13 | 0.36 | 0.04 | -0.02 | -0.17 |
| XVA 90-T | 0.63 | 0.32 | 0.24 | 0.03 | 0.44 | -0.14 | 0.01 | -0.15 | 0.05 | -0.08 | -0.07 | -0.25 | 0.22 |
| XVA below 90-T | 0.57 | 0.05 | -0.47 | 0.11 | 0.48 | 0.07 | -0.04 | -0.07 | 0.18 | 0.12 | 0.07 | -0.02 | 0.08 |
| XVA 30-1 | 0.39 | -0.03 | -0.15 | 0.59 | -0.36 | -0.38 | -0.11 | -0.08 | 0.07 | 0.32 | -0.03 | -0.18 | -0.05 |
| XVA 60-1 | 0.67 | -0.24 | -0.33 | 0.02 | 0.35 | -0.08 | 0.34 | 0.13 | -0.16 | -0.13 | -0.09 | 0.14 | -0.06 |
| XVA 90-1 | **0.77** | -0.32 | 0.07 | 0.40 | -0.12 | 0.08 | -0.01 | 0.13 | -0.02 | 0.19 | -0.07 | 0.09 | -0.13 |
| XVA below 90-1 | 0.51 | -0.43 | -0.04 | 0.45 | 0.02 | -0.13 | -0.23 | -0.15 | 0.09 | 0.09 | 0.36 | -0.01 | 0.03 |
| XVA 30-2 | 0.43 | 0.32 | -0.22 | 0.15 | **-0.57** | 0.21 | 0.42 | 0.07 | 0.15 | 0.04 | -0.07 | -0.10 | 0.13 |
| XVA 60-2 | -0.07 | 0.64 | 0.51 | 0.40 | 0.24 | -0.12 | -0.05 | 0.13 | 0.00 | 0.02 | 0.11 | 0.04 | -0.17 |
| XVA 90-2 | 0.38 | -0.50 | 0.55 | 0.05 | -0.10 | -0.01 | -0.14 | 0.01 | 0.02 | -0.02 | -0.37 | 0.10 | 0.18 |
| XVA below 90-2 | 0.36 | -0.52 | 0.63 | -0.16 | -0.11 | -0.08 | -0.05 | 0.00 | 0.19 | 0.09 | 0.18 | -0.08 | -0.07 |
| CCS 30-T | -0.10 | 0.38 | 0.03 | 0.32 | 0.16 | 0.49 | -0.30 | -0.22 | -0.33 | 0.02 | 0.03 | -0.31 | 0.15 |
| CCS 60-T | -0.30 | 0.03 | 0.04 | -0.28 | 0.03 | 0.46 | -0.30 | 0.44 | 0.14 | 0.37 | -0.14 | -0.02 | 0.17 |
| CCS 90-T | 0.06 | -0.53 | **0.67** | 0.22 | 0.12 | -0.17 | 0.13 | 0.19 | -0.05 | -0.07 | 0.12 | -0.02 | 0.15 |
| CCS below 90-T | -0.54 | -0.39 | 0.11 | 0.13 | 0.24 | -0.19 | 0.35 | 0.12 | 0.10 | 0.25 | 0.07 | 0.14 | 0.11 |
| CCS 30-1 | -0.15 | 0.32 | -0.10 | 0.46 | -0.55 | 0.15 | 0.23 | -0.03 | 0.34 | -0.19 | -0.15 | 0.08 | 0.07 |
| CCS 60-1 | 0.07 | -0.36 | 0.10 | 0.43 | 0.35 | 0.45 | -0.07 | 0.31 | 0.14 | -0.23 | 0.11 | 0.33 | -0.02 |
| CCS 90-1 | -0.37 | -0.20 | -0.09 | 0.59 | 0.29 | -0.19 | 0.09 | 0.30 | 0.05 | 0.06 | 0.14 | -0.14 | 0.13 |
| CCS below 90-1 | -0.51 | -0.44 | 0.18 | 0.13 | 0.19 | 0.04 | 0.42 | -0.05 | -0.16 | 0.25 | -0.17 | -0.04 | -0.06 |
| CCS 30-2 | -0.51 | 0.03 | 0.33 | 0.22 | -0.19 | 0.21 | 0.02 | 0.14 | 0.46 | -0.30 | -0.03 | 0.22 | 0.14 |
| CCS 60-2 | -0.66 | -0.32 | 0.10 | 0.24 | -0.02 | 0.32 | 0.08 | 0.05 | 0.10 | 0.29 | 0.14 | -0.06 | 0.00 |
| CCS 90-2 | -0.23 | -0.42 | -0.21 | 0.27 | 0.05 | 0.38 | -0.10 | -0.16 | 0.35 | -0.22 | 0.21 | -0.06 | 0.07 |
| CCS below 90-2 | -0.47 | -0.38 | -0.07 | 0.37 | 0.22 | 0.25 | 0.31 | -0.16 | -0.04 | 0.04 | -0.30 | -0.19 | -0.28 |
| CCF 30-T | -0.51 | 0.16 | -0.31 | -0.02 | -0.02 | 0.00 | -0.14 | -0.15 | -0.02 | **0.57** | 0.10 | 0.37 | 0.10 |
| CCF 60-T | 0.49 | -0.09 | 0.36 | -0.19 | -0.10 | 0.57 | -0.15 | -0.07 | -0.29 | 0.16 | 0.02 | 0.06 | 0.04 |
| CCF 90-T | 0.51 | 0.53 | 0.26 | 0.04 | 0.33 | 0.19 | 0.01 | -0.17 | -0.05 | -0.04 | -0.19 | 0.00 | 0.18 |
| CCF below 90-T | 0.48 | 0.20 | -0.16 | -0.03 | 0.19 | 0.12 | 0.29 | -0.40 | 0.28 | 0.05 | 0.30 | 0.22 | 0.20 |
| CCF 30-1 | 0.38 | 0.07 | -0.11 | **0.70** | -0.22 | -0.22 | -0.02 | 0.07 | 0.11 | 0.21 | 0.00 | -0.27 | 0.04 |
| CCF 60-1 | 0.44 | -0.21 | -0.51 | 0.03 | 0.18 | 0.33 | 0.12 | -0.02 | **-0.49** | -0.05 | 0.04 | 0.19 | -0.07 |
| CCF 90-1 | 0.69 | -0.24 | 0.25 | 0.32 | -0.14 | 0.24 | 0.10 | -0.01 | -0.09 | 0.07 | 0.05 | 0.27 | -0.18 |
| CCF below 90-1 | 0.50 | -0.17 | 0.07 | -0.07 | -0.41 | 0.10 | 0.04 | 0.03 | -0.40 | -0.19 | **0.48** | -0.10 | 0.01 |
| CCF 30-2 | 0.41 | 0.51 | 0.09 | 0.49 | -0.12 | 0.23 | 0.19 | 0.21 | -0.12 | 0.04 | -0.01 | 0.12 | 0.24 |
| CCF 60-2 | 0.05 | 0.56 | 0.38 | 0.37 | 0.35 | -0.12 | -0.18 | 0.11 | 0.00 | -0.23 | -0.04 | 0.25 | -0.13 |
| CCF 90-2 | 0.08 | 0.13 | 0.38 | -0.04 | 0.09 | -0.38 | **0.64** | 0.10 | -0.24 | 0.22 | -0.10 | 0.07 | 0.10 |
| CCF below 90-2 | -0.08 | 0.22 | 0.08 | -0.22 | -0.11 | -0.44 | -0.29 | **0.61** | -0.16 | -0.05 | 0.15 | 0.03 | 0.27 |
| Eigenvalue | 9.43 | 6.02 | 4.67 | 4.24 | 3.26 | 3.16 | 2.47 | 2.01 | 2.00 | 1.74 | 1.40 | 1.21 | 1.11 |
| Contributive ratio (%) | 19.64 | 12.54 | 9.73 | 8.84 | 6.79 | 6.58 | 5.16 | 4.19 | 4.17 | 3.63 | 2.92 | 2.53 | 2.32 |
| Cumulative contributive ratio (%) | 19.64 | 32.17 | 41.90 | 50.74 | 57.53 | 64.12 | 69.27 | 73.47 | 77.63 | 81.26 | 84.18 | 86.71 | 89.03 |


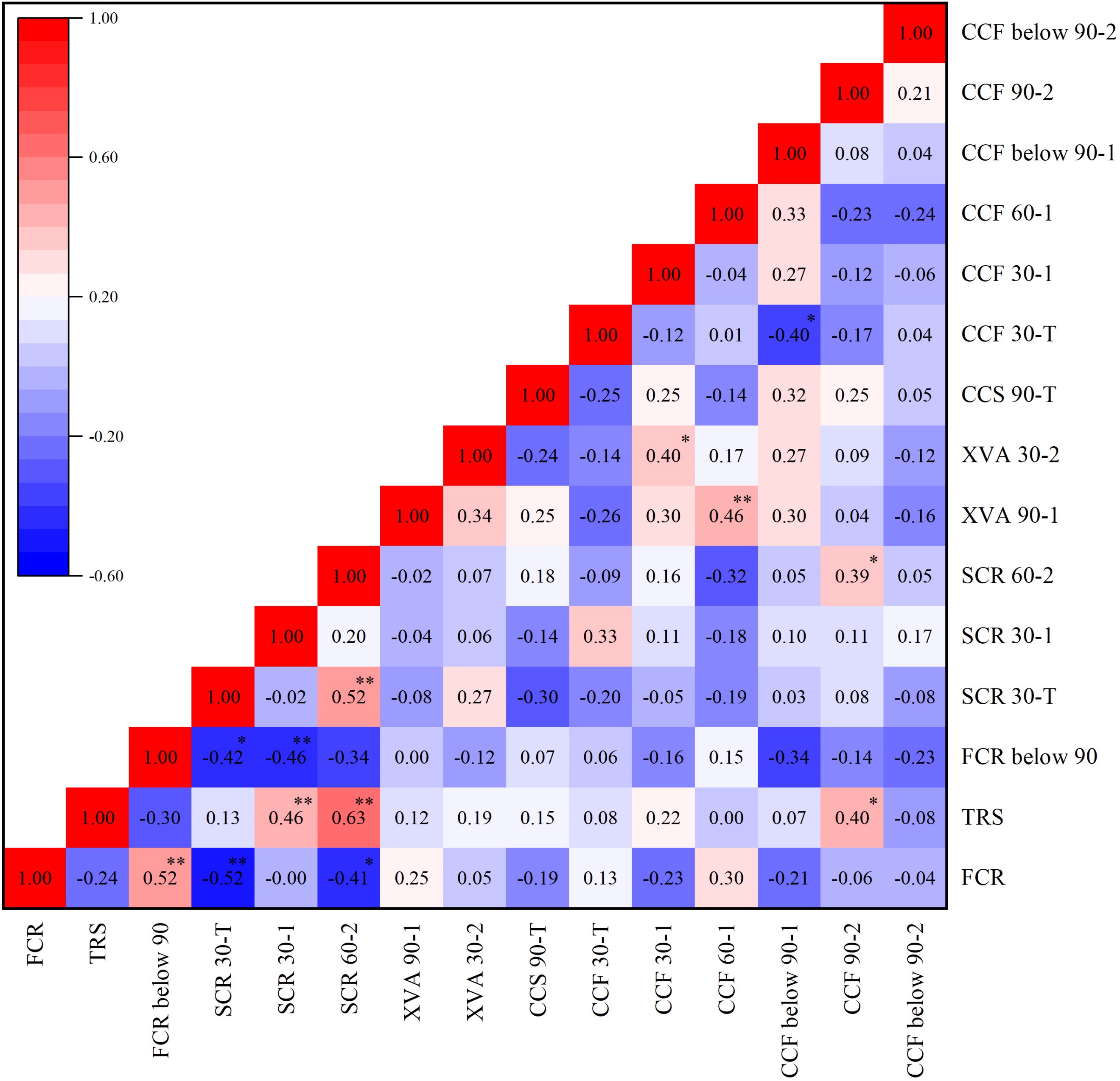


**Supplementary Figure 4.** Pearson correlation analysis among the root traits with the largest absolute loadings in each PC in the rhizoboxes experiment. *, ** and *** represent significant correlation at the levels of *P* < 0.05, *P* < 0.01 and *P* < 0.001, respectively. FCR: fine root length to coarse root length ratio; TRS: total root surface area; FCR below 90: fine root length to coarse root length ratio below 90 cm soil layer; SCR 30-T: total stele area to total cortical area ratio of the taproot in the 0-30 cm soil layer; SCR 30-1: total stele area to total cortical area ratio of the 1^st^ lateral root in the 0-30 cm soil layer; SCR 60-2: total stele area to total cortical area ratio of the 2^nd^ lateral roots in the 30-60 cm soil layer; XVA 90-1: xylem vessel area of the 1^st^ lateral root in the 60-90 cm soil layer; XVA 30-2: xylem vessel area of the 2^nd^ lateral roots in the 0-30 cm soil layer; CCS 90-T: cortical cell size of the taproot in the 60-90 cm soil layer; CCF 30-T: cortical cell files of the taproot in the 0-30 cm soil layer; CCF 30-1: cortical cell files of the 1^st^ lateral root in the 0-30 cm soil layer; CCF 60-1: cortical cell files of the 1^st^ lateral root in the 30-60 cm soil layer; CCF below 90-1: cortical cell files of the 1^st^ lateral root below 90 cm soil layer; CCF 90-2: cortical cell files of the 2^nd^ lateral roots in the 60-90 cm soil layer; CCF below 90-2: cortical cell files of the 2^nd^ lateral roots below 90 cm soil layer.


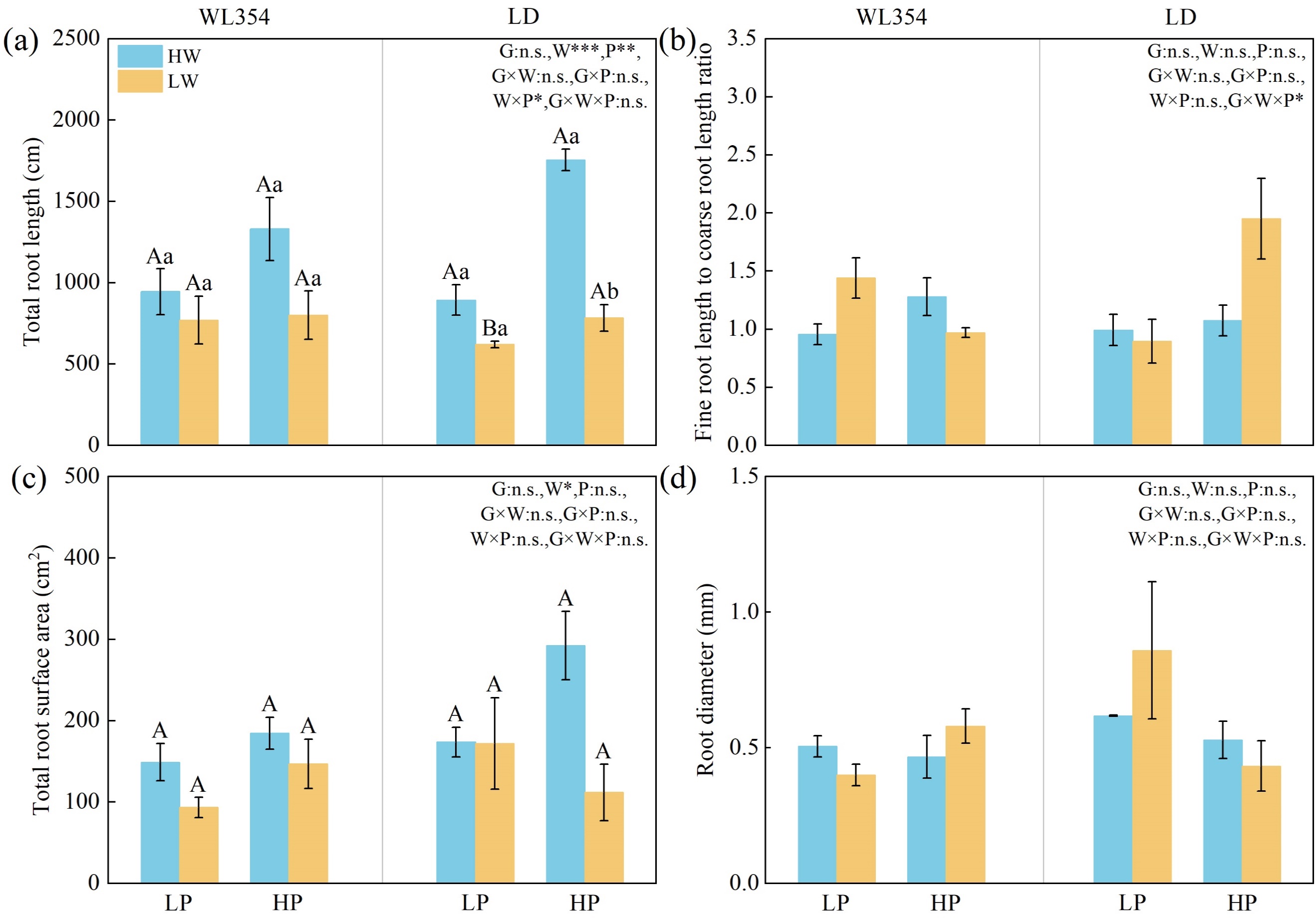


**Supplementary Figure 5.** Total root length (a), fine root length to coarse root length ratio (b), total root surface area (c), and root diameter (d) of WL354 and LD alfalfa under different water and phosphorus treatments in the field. Data represented as mean ± SE (n=3). When the three-way ANOVA indicated significant main effects of genotype, water, or P, differences within their respective treatment groups were analyzed. Different uppercases indicate significant differences between HW and LW treatments under the same genotypes and phosphorus treatments determined by independent samples t-test (*P* < 0.05); different lowercases indicate significant differences between LP and HP treatments under the same genotypes and water treatments determined by independent samples t-test (*P* < 0.05); *, ** and *** above the columns indicate significant difference between WL354 and LD under the same water and phosphorus treatments determined by independent samples t-test (*: *P* < 0.05, **: *P* < 0.01, ***: *P* < 0.001). HW: well-watered; LW: drought stress; HP: high P; LP: low P.


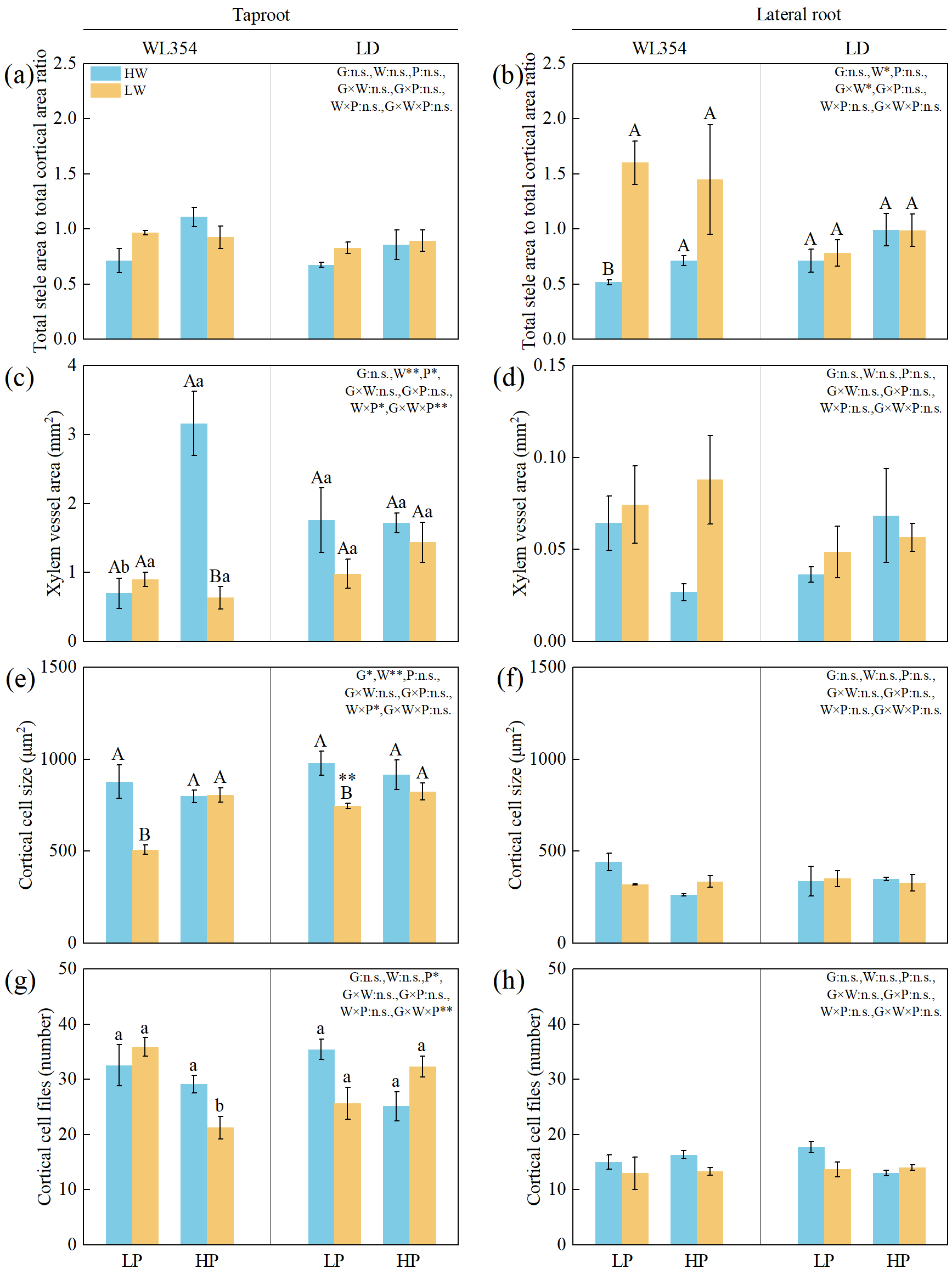


**Supplementary Figure 6.** Total stele area to total cortical area ratio (a and b), xylem vessel area (c and d), cortical cell size (e and f), and cortical cell files (g and h) of the taproot and lateral root of WL354 and LD alfalfa under different water and phosphorus treatments in the field. Data represented as mean ± SE (n=3). When the three-way ANOVA indicated significant main effects of genotype, water, or P, differences within their respective treatment groups were analyzed. Different uppercases indicate significant differences between HW and LW treatments under the same genotypes and phosphorus treatments determined by independent samples t-test (*P* < 0.05); different lowercases indicate significant differences between LP and HP treatments under the same genotypes and water treatments determined by independent samples t-test (*P* < 0.05); *, ** and *** above the columns indicate significant difference between WL354 and LD under the same water and phosphorus treatments determined by independent samples t-test (*: *P* < 0.05, **: *P* < 0.01, ***: *P* < 0.001). HW: well-watered; LW: drought stress; HP: high P; LP: low P.

**Supplementary Table 7.** Principal component (PC) analysis of root morphological traits in the field. The loading with the largest absolute value in each PC is bolded. PCs with eigenvalues > 1 are presented.

| Traits | PC1 | PC2 |
| --- | --- | --- |
| Total root length | 0.41 | **0.90** |
| Fine root length to coarse root Length ratio | -0.77 | 0.41 |
| Total root surface area | **0.91** | 0.38 |
| Root diameter | 0.78 | -0.51 |
| Eigenvalue | 2.19 | 1.39 |
| Contributive ratio (%) | 54.79 | 34.73 |
| Cumulative contributive ratio (%) | 54.79 | 89.52 |

**Supplementary Table 8.** Principal component (PC) analysis of root anatomical traits in the field. The loading with the largest absolute value in each PC is bolded. PCs with eigenvalues > 1 are presented.

| Traits | PC1 | PC2 | PC3 | PC4 |
| --- | --- | --- | --- | --- |
| Total stele area to total cortical area ratio of the taproot | -0.69 | 0.55 | 0.07 | -0.08 |
| Total stele area to total cortical area ratio of the lateral root | 0.07 | **0.76** | 0.38 | 0.07 |
| Xylem vessel area of the taproot | 0.72 | 0.03 | 0.12 | -0.35 |
| Xylem vessel area of the lateral root | 0.25 | 0.29 | 0.16 | **0.86** |
| Cortical cell size of the taproot | **-0.79** | -0.32 | 0.31 | 0.17 |
| Cortical cell size of the lateral root | -0.28 | -0.70 | 0.61 | -0.02 |
| Cortical cell files of the taproot | 0.56 | -0.51 | -0.12 | 0.36 |
| Cortical cell files of the lateral root | 0.52 | 0.12 | **0.73** | -0.16 |
| Eigenvalue | 2.36 | 1.83 | 1.19 | 1.06 |
| Contributive ratio (%) | 29.44 | 22.94 | 14.89 | 13.26 |
| Cumulative contributive ratio (%) | 29.44 | 52.38 | 67.27 | 80.52 |

**
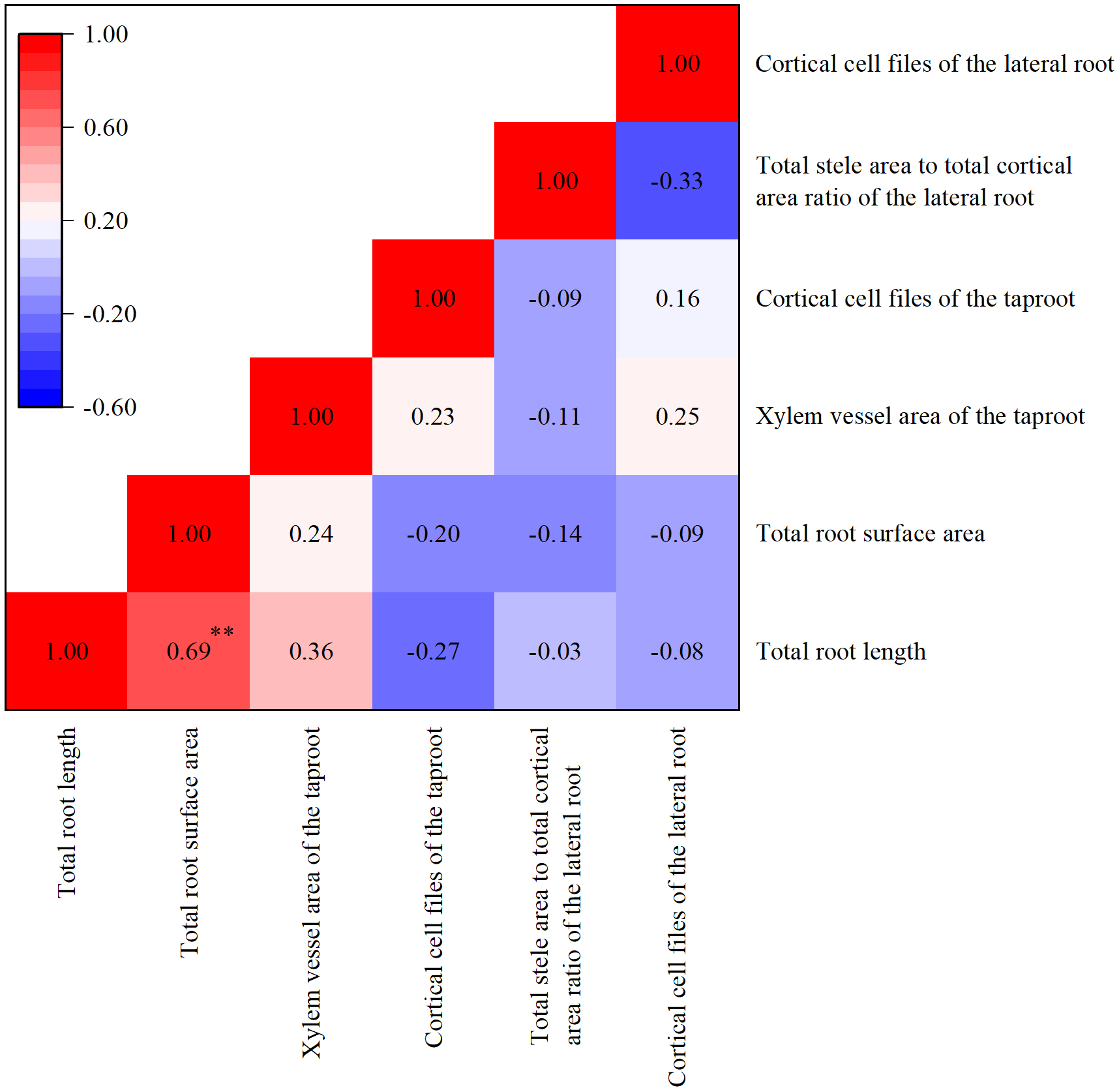
**

**Supplementary Figure 7.** Pearson correlation analysis among the root traits with the largest absolute loadings in each PC in the field. *, ** and *** represent significant correlation at the levels of *P* < 0.05, *P* < 0.01 and *P* < 0.001, respectively.
